# The critical role of MLKL in hemorrhagic stroke and the therapeutic potential of its associated protein network

**DOI:** 10.3389/fcell.2024.1509877

**Published:** 2025-01-20

**Authors:** Yi Wang, Moran Xu, Xiaoli Zuo, Sheng Wang, Yong Yu, Zhaobing Gao, Jingbo Qie, Ye Jiang, Fang Huang, Bingqing Xia

**Affiliations:** ^1^ Department of Translational Neuroscience, Jing’an District Centre Hospital of Shanghai, State Key Laboratory of Medical Neurobiology and MOE Frontiers Center for Brain Science, Institutes of Brain Science, Fudan University, Shanghai, China; ^2^ Stake Key Laboratory of Drug Research, Shanghai Institute of Materia Medica, Chinese Academy of Sciences, Shanghai, China; ^3^ Department of Digestive Diseases, Huashan Hospital, Institutes of Biomedical Sciences, Fudan University, Shanghai, China; ^4^ Department of Neurosurgery Zhongshan Hospital Fudan University, National Medical Center, Shanghai, China; ^5^ University of Chinese Academy of Sciences, Beijing, China; ^6^ Shanghai Fifth People's Hospital and Institutes of Biomedical Sciences, Fudan University, Shanghai, China; ^7^ Department of Neurosurgery, Minhang Hospital, Fudan University, Shanghai, China

**Keywords:** mlkl, ICH, hemorrhagic stroke, neuroinflammation, LC-MS/MS

## Abstract

**Introduction:**

Mixed Lineage Kinase Domain-Like Protein (MLKL), as the executor of necroptosis and a critical factor in the inflammation, has been shown to be associated with the progression of hemorrhagic stroke. Studies identified MLKL is a promoting factor in this process, suggesting its potential as a therapeutic target to mitigate posthemorrhagic stroke damage. However, the mechanisms by which MLKL functions in the process of intracerebral hemorrhage (ICH)-induced damage remain unclear.

**Methods:**

Here, we explored the correlation between MLKL and pathological damage in ICH patients through histopathological staining and RT-qPCR. Furthermore, we established an intracerebral hemorrhage model by collagenase IV injection in WT and *Mlkl^-/-^* mice. Subsequently, we investigated the impact of MLKL knockout on ICH pathological damage through behavioral tests, Western blotting, and RT-qPCR. Finally, we performed a proteomic analysis via LC-MS/MS to explore the potential interacting proteins of MLKL in the progression of ICH.

**Results:**

We found that MLKL is highly expressed in the brain tissue of ICH patients and is positively correlated with the extent of injury. However, we found that Mlkl knockout alone was insufficient to fully reverse neuroinflammation and pathological damage. Although Mlkl knockout has a limited effect on alleviating ICH damage, proteomics results indicate that MLKL can mitigate changes in proteins associated with inflammation, metabolism, and coagulation pathways, suggesting that MLKL may exert its effects through these pathways.

**Discussion:**

In summary, our results suggest that although MLKL is associated with the progression of ICH, single knockout of Mlkl is insufficient to fully reverse the pathological damage of ICH. Proteomic analysis indicates that co-targeting MLKL and its associated protein network may yield better therapeutic outcomes for hemorrhagic stroke.

## Introduction

Hemorrhagic stroke, which accounts for approximately 20% of all stroke cases, is most commonly manifested as intracerebral hemorrhage (ICH), presenting a critical challenge in clinical neurology due to its high morbidity and long-term disability (Montano et al., 2021). On one hand, primary ICH may result from prolonged, uncontrolled hypertension or cerebral amyloid angiopathy (CAA), which is more common in the elderly (Takebayashi and Kaneko, 1983; Viswanathan and Greenberg, 2011). On the other hand, secondary ICH may arise from vascular malformations, ruptured cerebral aneurysms, coagulation disorders, and other underlying conditions (Ruiz-Sandoval et al., 2006). The treatment of ICH primarily involves acute blood pressure management, reversal of coagulopathy, intracranial pressure and cerebral edema management, and prevention of secondary neurological injury (Cordonnier et al., 2018; Mayer and Rincon, 2005). However, apart from management in specialized neurocritical care units, there have been no specific therapeutic interventions that significantly improve outcomes following ICH. Thus, a comprehensive understanding of the pathological progression and molecular mechanisms underlying ICH, along with the identification of novel therapeutic targets, is crucial for advancing treatment strategies.

Research showed that targeting potential pathways such as S1PR and PD-1/PD-L1 to mitigate the inflammatory cascade surrounding ICH may improve patient outcomes (Tschoe et al., 2020). However, translating these potential targets into clinical practice remains challenging. Anyhow, these preclinical studies further suggest that during the progression of ICH, neuroinflammation worsen the severity of the condition. Besides, studies showed that neuronal death in the context of ICH exhibits characteristics of necroptosis (Zille et al., 2017). Activated RIPK3 mediates MLKL phosphorylation to execute necroptosis (Sun et al., 2012) or exacerbates neuronal death through the DAXX signaling pathway (Bai et al., 2024). Knocking out RIPK1 or MLKL, has been shown to alleviate brain edema, reduce neuronal death, decrease blood-brain barrier (BBB) permeability, and improve motor function (Lule et al., 2021). These findings support the central role of necroptosis in the pathogenesis of ICH. The contents released by necroptotic cells can further activate neuroinflammation, exacerbating pathological damage. Besides, Blood components such as bilirubin, hemoglobin, and hemin can directly activate immune cells (Lin et al., 2012; Loftspring et al., 2011; Loftspring et al., 2010). It has been found that NF-κB is activated in the brain tissue surrounding the hematoma, leading to the release of pro-inflammatory cytokines such as IL-1β and TNF (Teng et al., 2009). The brain’s resident macrophages, microglia, respond to heme, hemoglobin, and mitochondrial N-formyl peptides, promoting inflammation through the production of HMGB1 (high mobility group box 1), TNF, and IL-1β (Clausen et al., 2008; Ohnishi et al., 2011). During this process, various cytokines, including TNF, IL-6, IL-1β, IL-15, HMGB1, CSF-1 (colony-stimulating factor-1), and IL-17A, contribute to neurotoxic immune responses (Ohashi et al., 2023). These inflammatory mediators lead to BBB disruption, neuronal damage, and brain edema, further promoting neuronal apoptosis and impairing neuroplasticity, ultimately exacerbating neurological deficits. Pharmacological blockade of cytokines such as TNF can reduce BBB permeability, decrease brain edema, and improve behavioral outcomes following ICH (Lei et al., 2013; King et al., 2013). Therefore, mitigating neuronal death and neuroinflammation through therapeutic interventions may hold potential in treating ICH and improving outcomes after hemorrhagic stroke.

MLKL, as the executor of necroptosis, has been established as a key player in processes such as necroptosis and inflammation (Sun et al., 2012; Wang et al., 2014; Conos et al., 2017). Enhancing MLKL’s channel function has been shown to exacerbate necroptosis and inflammation in BV2 cells, suggesting that MLKL could serve as a potential target for alleviating neuroinflammation (Xia et al., 2022a). Indeed, several studies have highlighted the role of MLKL in the progression of ICH. In a rat model created by injecting collagenase IV into the right basal ganglia, the phosphorylation of MLKL was observed, and the use of the RIPK3 inhibitor GSK872 effectively reduced MLKL phosphorylation, thereby mitigating pathological damage and behavioral deficits (Huang et al., 2022). Celastrol has also been shown to inhibit RIPK3/MLKL pathway-mediated necroptosis, providing neuroprotection by alleviating blood-brain barrier disruption (Xu H. et al., 2021). Moreover, direct knockout of RIPK1 or MLKL has been found to alleviate ICH-induced damage (Lule et al., 2021). In summary, MLKL is a potential target for alleviating ICH. However, its mechanisms remain unclear and are subject to debate. Therefore, a systematic exploration of the role of MLKL in the process of ICH-induced damage, along with elucidating its influencing protein networks, is of significant importance. Here, we further explored the feasibility of MLKL as a therapeutic target for mitigating ICH and investigated the protein networks associated with MLKL in this context.

## Results

### MLKL expression correlates with disease progression in ICH patients

**FIGURE 1:**
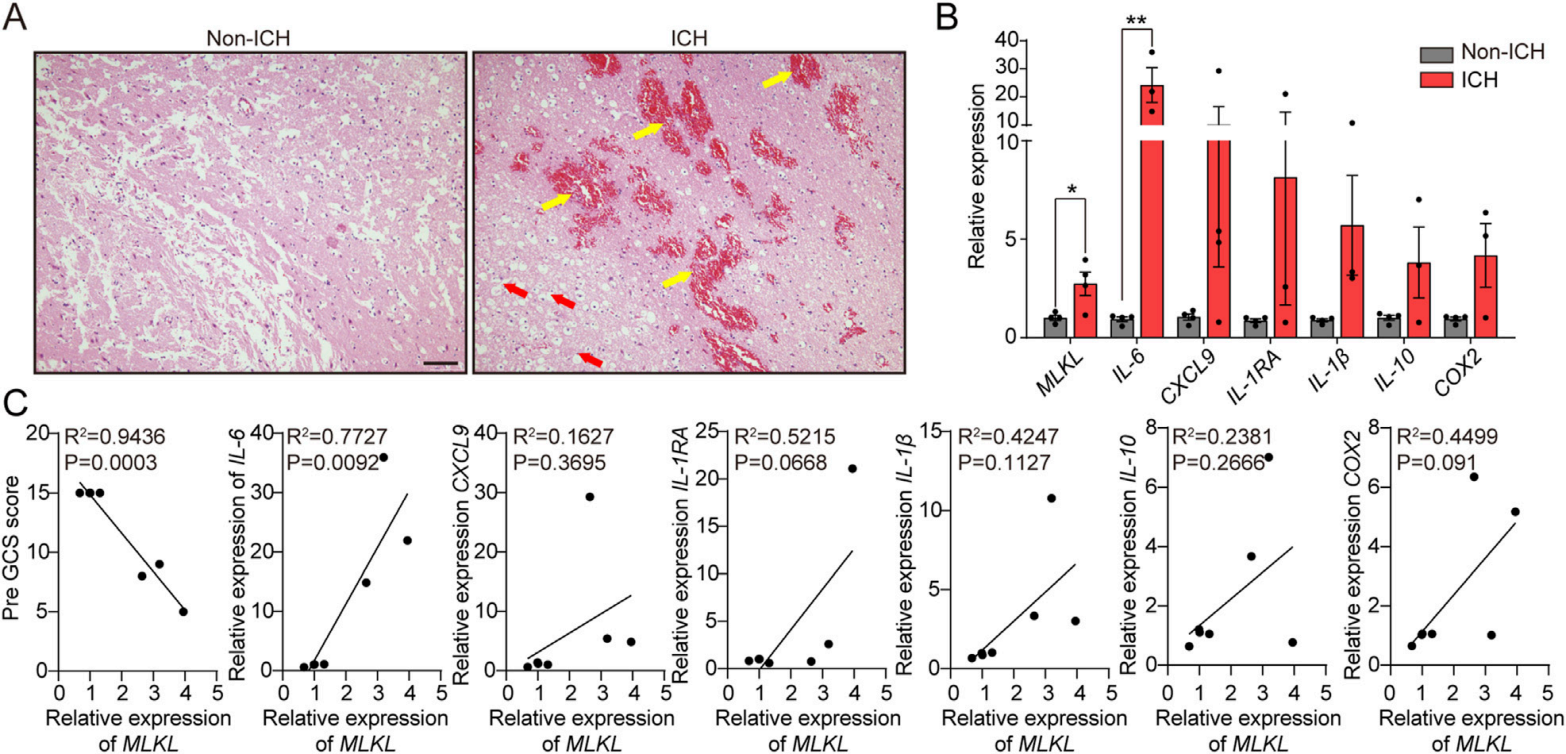
MLKL Correlation with ICH Progression in Patients **(A)** Pathological damage sections of brain tissue from ICH patients and non-ICH patients. Scale bar 100 μm. **(B)** RT-qPCR analysis of brain tissue from ICH patients and non-ICH patients. **(C)** Correlation analysis of MLKL expression levels with Pre-GCS score and inflammation-related protein expression levels.

To confirm the clinical relevance of MLKL in ICH, we analyzed pathological damage and protein expression in ICH patients (Tables 1, 2). First, brain tissues from ICH patients and normal controls were subjected to H&E staining (Figure 1A). No neuronal damage was observed in normal brain tissues. In contrast, ICH patients were observed small venules exhibiting perivenular bleeding with extravasated erythrocytes and intracerebral hemorrhage (Yellow arrow) hemorrhage associated to perivascular infiltrate of neutrophils, vacuolating neuronal (neuronal Vacuolation) increased (Red arrow). RT-qPCR results showed that, compared to the control group, ICH patients exhibited elevated expression of pro-inflammatory cytokines such as IL-1β and IL-6, as well as the chemokine CXCL9, indicating intense neuroinflammation in ICH patients. Moreover, MLKL was highly expressed in the brain tissues of ICH patients, suggesting its potential involvement in ICH progression (Figure 1B). Correlation analysis shows that MLKL expression is negatively correlated with the Pre-GCS score and positively correlated with pro-inflammatory cytokines and chemokines (Figure 1C).

**TABLE 1:** Clinical characteristics of ICH patients.

	1	2	3	4	5
Age	45	61	66	76	54
Sex	Male	Female	Male	Male	Female
Medical history					
Hypertension	N	Y	Y	Y	Y
Diabetes mellitus	Y	Y	Y	Y	Y
Hyperlipidaemia	N	Y	Y	Y	N
Heart disease	N	Y	Y	Y	N
Ever smoking	N	N	N	N	Y
Alcoholism	Y	N	N	N	Y
ICH event					
Pre GCS score	8	9	5	7	7
Systolic blood pressure, mmHg	180	165	170	190	180
Diastolic blood pressure, mmHg	90	100	90	100	90
Post GCS score	12	15	12	11	15
Hospitalization days	14	15	16	19	13

**TABLE 2:** Clinical characteristics of *None-ICH patients*.

	1	2	3	4	5
Age	34	45	43	36	65
Sex	Male	Female	Female	Female	Male
Medical history					
Hypertension	N	N	N	N	N
Diabetes mellitus	N	N	N	N	Y
Hyperlipidaemia	N	N	N	N	N
Heart disease	N	N	N	N	N
Ever smoking	N	Y	N	N	Y
Alcoholism	N	N	Y	N	N
Pathological results	pituitary adenoma	meningioma	pituitary adenoma	pituitary adenoma	meningioma
Hospitalization days	8	9	9	9	10
Pre GCS score	15	15	15	15	15
Systolic blood pressure, mmHg	90∼139	90∼139	90∼139	90∼139	90∼139
Diastolic blood pressure, mmHg	60∼89	60∼89	60∼89	60∼89	60∼89

### *Mlkl* knockout exhibits limited efficacy in alleviating ICH damage

**FIGURE 2:**
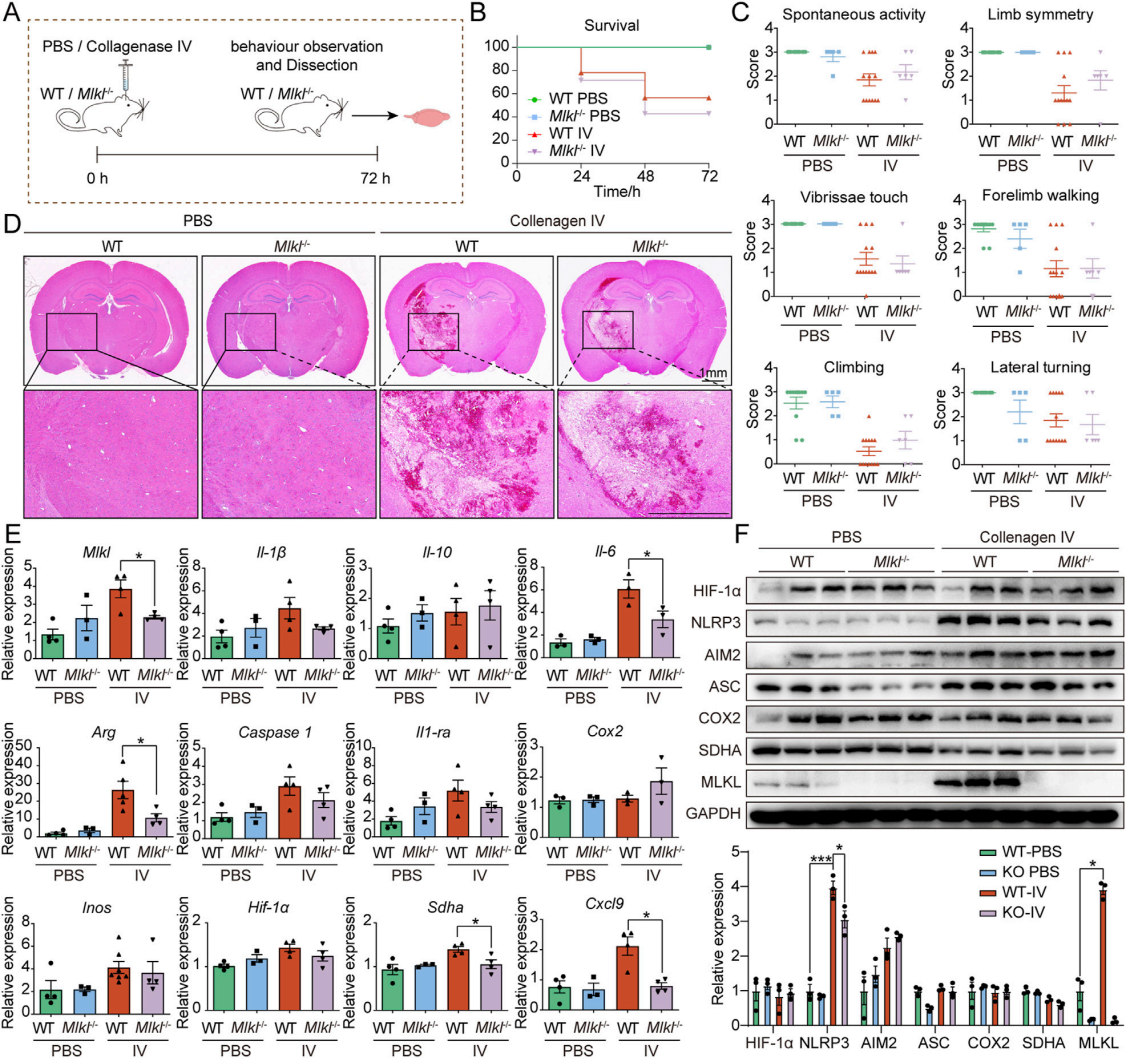
Limited efficacy of *Mlkl* knockout in alleviating ICH damage **(A)** Flowchart of the mouse intracerebral hemorrhage model. **(B)** Survival curves for WT and Mlkl−/− mice in the intracerebral hemorrhage model. **(C)** Neurological behavioral assessments in mice. **(D)** Pathological sections of brain tissue from WT and Mlkl−/− mice. **(E)** RT-qPCR analysis of brain tissue from WT and *Mlkl* ^−/−^ mice. **(F)** Upper, Western blot analysis of brain tissue from WT and *Mlkl* ^−/−^ mice, lower, statistical analysis of gray values from the upper panel.

To further investigate the role of MLKL in ICH, we generated an ICH model by injecting collagenase IV into the striatum of WT and *Mlkl* ^−/−^ mice (Figure 2A). We used CRISPR-Cas9 technology to delete 10 bp in the coding sequence of the *Mlkl*, resulting in a frameshift in the mRNA codons, the appearance of a premature stop codon, and early termination of translation (Supplementary Figure S1). Upon assessing the survival rate within 72 h post-collagenase IV injection, we found that *Mlkl* ^−/−^ mice exhibited a similar survival rate compared to WT mice (Figure 2B). Next, we evaluated the neurological behavior of the mice post-collagenase IV injection, including spontaneous activity, limb symmetry, vibrissae touch, forelimb walking, climbing, and lateral turning. The results showed that mice injected with collagenase IV demonstrated impaired behavioral performance compared to the sham group (Figure 2C). Knockout of MLKL resulted in a limited improvement in spontaneous activity, limb symmetry, and climbing abilities, but did not affect the scores for vibrissae touch, forelimb walking, and lateral turning (Figure 2C). We further evaluated the pathological damage in the model mice. H&E staining (Figure 2D) did not show damage in the sham group, while cells and extracellular matrix around the injury zone were disorganized after ICH. It was also observed that *Mlkl* ^−/−^ ICH mice ameliorated the neuronal disorganization and decreased the hemorrhage area in the brain tissue. *Mlkl* knockout did not appear to significantly alleviate the pathological damage associated with ICH. Further RT-qPCR analysis revealed that *Mlkl* knockout moderately reduced the expression of pro-inflammatory proteins such as *Arg*, *Il-6* and *Sdha*, but had no effect on the expression of *Caspase1*, *Cox2*, *Inos* and *Hif-1α*. Finaly, we examined protein expression changes following *Mlkl* knockout. Consistent with the findings in human brain tissues, collagenase-induced ICH significantly upregulated MLKL expression. Additionally, collagenase injection promoted the expression of pro-inflammatory proteins such as MLKL, NLRP3 and AIM2. *Mlkl* knockout partially reduced NLRP3 expression but had no effect on other pro-inflammatory proteins. Summarizing these findings, in the collagenase IV-induced ICH model, *Mlkl* knockout reduced the expression of certain pro-inflammatory proteins, but did not significantly alleviate the pathological damage caused by ICH.

### Proteomics reveal *Mlkl* knockout insufficient to reverse neuroinflammation during ICH progression

**FIGURE 3:**
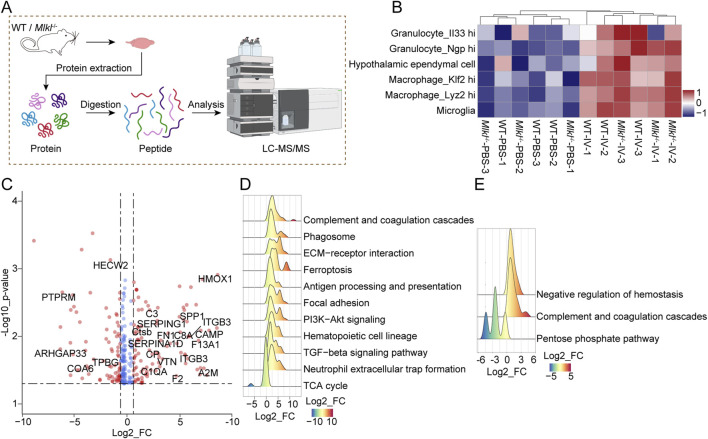
Quantitative proteomics suggested MLKL knock out is insufficient to reverse neuroinflammation during ICH progression. **(A)** Flowchart of proteomics analysis. **(B)** Unsupervised hierarchical clustering and GSVA score of indicated immune cells for the proteome of WT-IV model and the control group based on database Mouse Cell Atlas (MCA, http://bis.zju.edu.cn/MCA/atlas.html). cells representing GSVA scores are coded by gradually varied colors. **(C)** The volcano plots for the comparison between proteome patterns of brain tissues treated with PBS or collagenase IV in the WT mice. Proteins with significant changes (*p* < 0.05, bilateral Student’s t test) are colored with red (fold change ≥1.5). **(D)** Ridge plot for GSEA of the proteome of IV model and control group of WT mouse based on mouse GOBP database (FDR <0.05, Permutation test, Benjamini-Hochberg (BH)-adjusted *p*-value). Protein Fold Change values are represented and coded by *x*-axis and gradually varied colors. **(E)** Ridge plot for GSEA of the proteome from collagenase IV-treated WT and *Mlkl* ^−/−^ mice based on mouse GOBP database (FDR <0.05, Permutation test, Benjamini-Hochberg (BH)-adjusted *p*-value). Protein Fold Change are represented and coded by *x*-axis and gradually varied colors.

Our results indicate that *Mlkl* knockout does not effectively mitigate ICH damage, despite our other experiments and previous studies suggesting that MLKL is associated with ICH progression. The results suggest that targeting MLKL alone may not be sufficient to reverse the severe damage in models like ICH, and identifying novel therapeutic targets for combination therapy may yield better outcomes. Given that *Mlkl* knockout partially alleviated neuroinflammation, we next devoted to identify potential new targets by exploring the protein networks influenced by MLKL during ICH progression. To this end, we performed the LC-MS/MS analysis on control and collagenase IV-treated brain tissues of WT or *Mlkl* ^−/−^ mice (Figure 3A; Supplementary Table S1). Hierarchical clustering revealed the distinct expression profile between the collagenase IV-treated and control groups, which was not affected by *Mlkl* knockout (Figure 3B). Besides, gene set variation analysis (GSVA) showed that *Mlkl* knockout had no effect on immune cell pattern in group IV, further indicating that MLKL deficiency alone is insufficient to fully alleviate neuroinflammation during ICH progression (Figure 3B). GSVA scores determined by R package GSVA based on sc-RNA seq dataset in Mouse Cell Atlas (Han et al., 2018) were coded by gradually varied colors and assigned to indicated cells.

Direct comparison between protein profiles of collagenase IV-treated and control groups of WT mouse revealed 178 differentially expressed genes, including proteins participating in inflammation process, such as CD34/36, CSF1R, C3, VWF, and integrin proteins, etc (Figure 3C). Meanwhile, gene set enrichment analysis (GSEA) on the global proteome pattern showed that blood coagulation and immune-related pathways were significantly upregulated in the collagenase IV-treated groups (Figure 3D). The GSEA based on comparison between the proteome data of brain tissue in WT *versus* *Mlkl* ^−/−^ mice after collagenase IV treatment revealed the negative regulation of hemostasis inversely (Figure 3E). The results suggested that the blood coagulation, which may be controlled by MLKL, is closely associated with ICH progression. The coagulation system forms blood clots to seal ruptured vessels, preventing further bleeding and playing a hemostatic role. However, dysregulated coagulation responses may exacerbate the severity of the disease. Notably, previous studies found that Tenuigenin (TNG) has potential therapeutic effects on ICH and is closely associated with the complement and coagulation cascades (Wang et al., 2024).

Based on the above results, we speculate that MLKL is an important contributor to the progression of ICH. However, for reasons currently unknown to us, *Mlkl* knockout alone is insufficient to fully alleviate neuroinflammation and pathological damage during ICH progression. Therefore, we hypothesize that jointly targeting the MLKL-associated protein network may better enhance the pharmacological effects and subsequently mitigate pathological damage.

### Proteomics reveals the MLKL-influenced protein network and potential new targets during ICH progression

**FIGURE 4:**
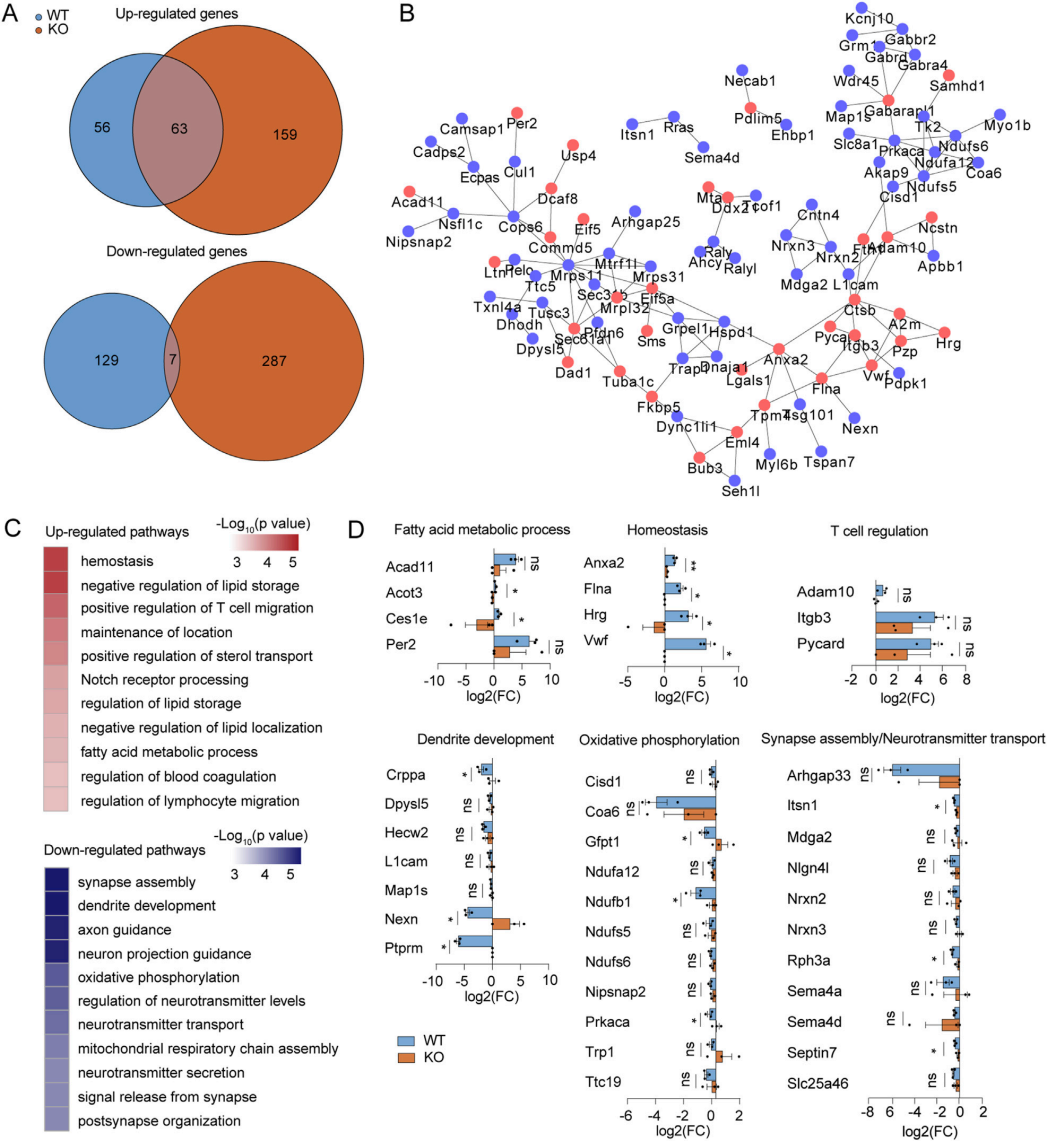
Proteomic screen identifies functional cofactors for MLKL participating in IV development. **(A)** Venn diagrams summary of the numbers of upregulated (up) or downregulated genes (down) by collagenase IV treatment in the WT (blue) or *Mlkl* ^−/−^ littermates (red), comparing with the normal controls, respectively. **(B)** PPI network of upregulated (blue nodes) or down-regulated (red nodes) proteins in blue areas in **(A)**. **(C)** The representative function enrichment terms of upregulated or downregulated genes in blue areas in **(A)**. Functional terms were labeled and color-coded with *p*-value (Fisher’s exact test) according to the legend. **(D)** Differences of fold changes in expression levels of indicated genes induced by collagenase IV stimulation (comparing with the normal control) between WT mice (blue) and *Mlkl* ^−/−^ littermates (red). The genes are annotated for the pathways they belong to.

Subsequently, we investigated the impact of *Mlkl* knockout on protein expression in the brain tissue of mice following collagenase IV treatment. We hypothesized that those proteins whose expression is significantly regulated by collagenase treatment in the wild type, but not correspondingly altered in *Mlkl* knockout mice, may be synergistic molecules of MLKL that work together to influence the ICH progression. In the proteome dataset, the expression of 56 proteins and 129 proteins were significantly upregulated and downregulated (|fold change| ≥ 1.5, *p* < 0.05, bilateral Student’s *t*-test) by collagenase IV-treated brains comparing with the normal brains in WT mice respectively, but not altered likewise in the *Mlkl* ^−/−^ littermate (Figure 4A). We constructed the MLKL-mediated PPI network based on String database, the nodes in the network are proteins whose expression is significantly regulated by collagenase treatment in the wild type, but not correspondingly altered in *Mlkl* knockout mice (Figure 4B). These proteins form a closely interconnected network, suggesting that MLKL may act as a key node regulating inflammation-related pathways. Specifically, the activation of proteins participating in fatty acid metabolic process (including Acad11, Acot3, Ces1e, Per2), Homeostasis (Anxa2, Flna, Hrg, Vwf) and T cell regulation (Adam10, Itgb3, Pycard) were observed in collagenase IV-treated brains of WT mouse rather than *Mlkl* ^*−/−*^ mouse (Figures 4C, D). Homeostasis and fatty acid metabolism changes are critical events during inflammation and ICH progression (Hu et al., 2021; Garton et al., 2016; Xia et al., 2022b). Studies discovered that proteins involved in fatty acid metabolism may serve as a potential target to mitigate ICH-induced white matter injury (Zhang et al., 2024). Furthermore, T cells are closely associated with neuroinflammation during ICH progression. Published researches have shown that T cell immunoglobulin and mucin domain-3 (Tim-3) plays a role in post-ICH neuroinflammation (Xu et al., 2018), with early elevation in ICH models correlating positively with TNF-α and IL-1β levels as well as brain water content. Knockout of Tim-3 has been shown to effectively alleviate ICH (Xu et al., 2013).

On the other hand, the proteins related to dendrite development (Crppa, Dpysl5, Hecw2, etc.), oxidative phosphorylation (Cisd1, Coad6, Gfpt1, etc.), and nervous system development or function (Arhgap33, Nrxns, Semas, etc.) were suppressed by collagenase IV treatment in WT mice but not in *Mlkl* ^−/−^ mice (Figures 4C, D). The reduction in oxidative phosphorylation and tricarboxylic acid (TCA) cycle function is a hallmark of M1 macrophage polarization and the inflammatory process (Jha et al., 2015). Studies showed that variations in oxidative phosphorylation-related genes may increase the risk of ICH (Anderson et al., 2013). Pathological damage caused by ICH can impair dendrite development, nervous system development, and function, which is also reflected in the observed proteomic changes. *Mlkl* knockout effectively reduced the upregulation of proteins related to fatty acid metabolic processes, homeostasis, and T cell regulation, while alleviating the downregulation of proteins associated with oxidative phosphorylation, dendrite development, and nervous system development or function. These findings suggest that MLKL may exacerbate neuroinflammation and pathological damage in ICH through these pathways.

## Discussion

Although numerous studies have demonstrated the critical role of MLKL in the progression of hemorrhagic stroke (Huang et al., 2022; Xu H. et al., 2021), there is currently no evidence to suggest that MLKL can serve as a drug target for alleviating hemorrhagic stroke. In this study, we found that *Mlkl* knockout alone was insufficient to fully reverse neuroinflammation and pathological damage during ICH progression. Additionally, we found that *Mlkl* knockout did not significantly affect the necroptosis pathway. While the knockout of MLKL inhibited neuronal necroptosis during ICH, other forms of cell death, such as ferroptosis (Lv et al., 2024; Li et al., 2017), could still occur. Studies have shown that the activation of RIPK1 and MLKL during ICH exhibits cell-type specificity. RIPK1 is activated early in neurons, endothelial cells, and pericytes, but not in astrocytes. In contrast, MLKL is activated in astrocytes and neurons but not in endothelial cells and pericytes, indicating that RIPK1 mediates cell necrosis in a partially MLKL-dependent manner (Lule et al., 2021). Other studies have indicated that RIPK3 may promote necrosis via the CaMKII and AIF pathways (Xu Y. et al., 2021). Moreover, although MLKL has been identified as a key factor in the neuroinflammatory process, the deficiency of MLKL could not fully prevent the occurrence of neuroinflammation. Compensatory mechanisms or other pro-inflammatory pathways may play a significant role in this process, mediating the persistent damage following ICH. Given that MLKL is indeed a critical factor in the progression of ICH, we hypothesize that co-regulating the MLKL-associated protein network could lead to improved therapeutic outcomes.

For the first time, we revealed the impact of *Mlkl* knockout on protein expression during ICH progression. Our findings indicated that *Mlkl* knockout decreased the upregulation of proteins related to fatty acid metabolism, homeostasis, and T cell regulation, while alleviating the downregulation of proteins involved in oxidative phosphorylation, dendrite development, and nervous system function, suggesting that MLKL may act through these pathways. Consistent with our findings, metabolomic analysis revealed that the fatty acid metabolite 20-hydroxy-leukotriene B4 (20-OH-LTB4) and its key enzyme, cytochrome P450 family 4 subfamily F member 2 (CYP4F2), could serve as potential biomarkers for ICH diagnosis (Zhang et al., 2021). Disruption of homeostasis in the cerebrospinal fluid plays a critical role during the progression of ICH, and reducing brain edema while mitigating homeostatic disruption is a key aspect of post-hemorrhagic stroke care (Wan et al., 2023). Transcriptomic analysis of ICH models has also revealed similar conclusions, with multiple T cell-specific genes being enriched (Durocher et al., 2021). Impairment of oxidative phosphorylation is a hallmark event in the inflammatory process. Studies found that downregulation of UCP2 exacerbates the uncoupling of oxidative phosphorylation, intensifying neuroinflammation in BV2 microglia and mouse models of brain hemorrhage, suggesting that endogenous UCP2 may inhibit neuroinflammation following hemorrhagic stroke, thereby providing neuroprotective effects (Yan et al., 2022). Furthermore, the damage to dendritic development and nervous system function observed during the ICH process was alleviated in *Mlkl* knockout mice, indicating that the associated proteins may play a role in mitigating ICH-induced damage. Therefore, we hypothesize that co-targeting the MLKL-associated protein network may further improve therapeutic outcomes for hemorrhagic stroke. However, the limitations of our study include the lack of exploration into the feasibility of these pathway proteins as therapeutic targets for ICH. Additionally, due to the absence of clinical omics data, we were unable to compare the proteomic changes in ICH mice with those of human patients. These potential targets and pathways require further investigation in future studies.

In summary, our study revealed the proteomic changes during ICH progression, with a particular focus on the impact of *Mlkl* knockout on the associated protein networks. Based on these findings, we have identified potential therapeutic targets that may be co-targeted with MLKL, providing new insights for the development of improved treatment strategies for ICH.

## Methods

### Clinical sample collection

This study was approved by the Minhang Hospital, Fudan University. Informed consent was obtained from all the patients and/or their relatives. All patients underwent surgical treatment. The inclusion criteria for ICH patients were: 1) a confirmed diagnosis of acute cerebral hemorrhage based on clinical symptoms and imaging (CT); 2) aged 18–80 years; and 3) admitted within 24 h of hemorrhagic stroke onset. The inclusion criteria for none ICH patients were:1)Pathological diagnosis of meningioma or pituitary tumor patients; 2) aged 18–80 years. Exclusion criteria included history of hemorrhagic stroke, concurrent inflammatory diseases, and ongoing immunosuppressive therapy. Control subjects were obtained from the department of neurosurgery, Minhang Hospital, Fudan University. Demographic data, including age, gender, and relevant medical history, were recorded.

### ICH mouse model

Before the surgery, isoflurane (R510-22-10, RWD, China) was administered via an animal anesthesia machine (MSS-3S, Renyi Instrument, China) to induce anesthesia in mice. Adjust the concentration on the anesthesia machine to 4% to induce anesthesia in the mice. Once full anesthesia was achieved, the mice were secured in a stereotaxic frame. The isoflurane concentration was then adjusted to 2% to maintain a stable anesthetic state throughout the surgery. Collagenase Ⅳ (C5138, Sigma, United States) or PBS was injected into the center of the striatum of mice at 2.0 mm lateral to bregma, and 3.5 mm deep relative to dura. A volume of 0.5 μL of collagenase Ⅳ (0.12 U/μL) or PBS was slowly injected into the striatum at 0.25 μL/min. The needle was kept for another 5 min after the infusion to prevent Collagenase Ⅳ leakage. After surgery, the mice were under observation with free access to water and food. At 72 h, all mice were euthanized by intraperitoneal injection of 60 mg/kg anesthetic (Zoletil^®^50, Virbac, France), followed by tissue collection.

### Neurobehavioral test

Neurobehavioral functions were evaluated by an independent researcher blinded to the procedure by the Garcia test and forelimb placement test (Yu et al., 2017; Manaenko et al., 2011). Neurofunctional tests were conducted in a blinded fashion prior to euthanasia, 72 h after surgery. This composite assessment consists of six independent sub-tests evaluating spontaneous activity (I), vibrissae proprioception (II), limb symmetry (III), lateral turning (IV), forelimb walking (V) and climbing (VI). Performance and evaluation of each sub-test are described in the following paragraph. Spontaneous activity: Animals were observed for 5 min in their normal environment. Scores indicate the following: 3, mouse approached at least 3 walls of the cage; 2, mouse approached less than 3 walls; 1, mouse barely moved; 0, mouse did not move at all. Vibrissae proprioception: A cotton swap was moved from the rear of the animal towards its head, touching the vibrissae gently on each side at a time. Scores indicate the following: 3, mouse equally turned head on both sides; 2, asymmetric response; 1, missing respond on one side. Limb symmetry: The mouse was suspended by the tail to assess movement of the limbs. Scores indicate the following: 3, all limbs were extended symmetrically; 2, asymmetric extension; 1, limbs on one side showed minimal movement; 0, hemiplegia (no limb movement). Lateral turning: The mouse was suspended by the tail and a blunt stick was moved along each side of the body causing lateral turning towards the stimulus. Scores indicate the following: 3, animal turns at least 45° on both sides; 2, animal turns equally to both sides but less than 45°; 1, unequal turning; 0, no turning at all. Forelimb walking: The mouse was suspended by its tail allowing both forepaws to touch the edge of a table. Scores indicate the following: 3, forelimbs were equally outstretched and the animal walked symmetrically on forepaws; 2, asymmetric outstretch and impaired forepaw walking; 1, minimal movement of one forelimb; 0, hemiplegia (no limb movement). Climbing: The mouse was placed on a rough surface (22 × 44 cm) at a 45° angle with the table. Scores indicate the following: 3, mouse climbed to the top of the surface; 2, asymmetric or impaired climbing; 1, animal failed to climb or showed tendency of circling.

### Histology

The whole brain of the model mice or brain tissue from patients was fixed in 4% PFA, followed by paraffin embedding. The tissue blocks were then sectioned into 3 μm slices according to standard procedures. The sections were stained with hematoxylin and eosin (H&E), and examined by light microscopy (Leica DM 6B, Leica, Germany).

### RT-qPCR

Total RNA samples were extracted from tissues using Trizol (Invitrogen, United States), following the manufacturer’s instruction. The experiment was performed at least three times using SYBR Green Master Mix (11184ES03, Yeason, China) to quantify the mean values of delta Ct and SEM (Standard Error of Mean). The primers used for quantification were listed in Table 3.

**TABLE 3:** List of Quantitative Real-time PCR (qRT-PCR) primers.

Primer name	FWD sequence	REV sequence
Mouse *Il-10*	ATA​ACT​GCA​CCC​ACT​TCC​CA	GGG​CAT​CAC​TTC​TAC​CAG​GT
Mouse *Il-6*	AGT​TGC​CTT​CTT​GGG​ACT​GA	TCC​ACG​ATT​TCC​CAG​AGA​AC
Mouse *Il-1β*	GAA​GTT​GAC​GGA​CCC​CAA​AA	CCA​CAG​CCA​CAA​TGA​GTG​ATA​C
Mouse *Tnf-α*	TCG​TAG​CAA​ACC​ACC​AAG​TG	GGA​GTA​GAC​AAG​GTA​CAA​CCC​A
Mouse *Inos*	GTT​CTC​AGG​CCA​ACA​ATA​CAA​GA	GTG​GAC​GGG​TCG​ATG​TCA​C
Mouse *Arg1*	ACC​TGG​CCT​TTG​TTG​ATG​TC	ACT​GCC​AGA​CTG​TGG​TCT​CC
Mouse *Hmgb1*	CTC​CTT​CGG​CCT​TCT​TCT​TGT	TCT​CAT​AGG​GCT​GCT​TGT​CA
Mouse *Gapdh*	AAC​TTT​GGC​ATT​GTG​GAA​GG	ACA​CAT​TGG​GGG​TAG​GAA​CA
Mouse *Sdha*	CTG​GGA​GCA​TGG​ACG​AAT​TG	CGG​CCA​ATC​TTG​CAG​TAC​TC
Mouse *Hif-1α*	TCA​GCA​TAC​AGT​GGC​ACT​CA	AAG​GGA​GCC​ATC​ATG​TTC​CA
Mouse *Cox2*	AGA​AGG​AAA​TGG​CTG​CAG​AA	GCT​CGG​CTT​CCA​GTA​TTG​AG
Mouse *Cxcl9*	GAA​CGG​AGA​TCA​AAC​CTG​CC	CGA​CGA​CTT​TGG​GGT​GTT​TT
Mouse *Il1-ra*	CCA​GCT​CAT​TGC​TGG​GTA​CT	TTC​TCA​GAG​CGG​ATG​AAG​GT
Mouse *Ccl12*	GCT​ACC​ACC​ATC​AGT​CCT​CA	GGG​TCA​GCA​CAG​ATC​TCC​TT
Human *Mlkl*	AGG​ACG​TGA​ACA​GGA​AGC​TG	AGG​ACG​CTC​CTT​GGC​TTA​TG
Human *Arg1*	ACC​AGG​GTT​CAT​CAG​AGG​TG	GCC​CCG​TGA​GTT​CGT​ATT​TC
Human *Il-6*	TGC​AAT​AAC​CAC​CCC​TGA​CC	ATT​TGC​CGA​AGA​GCC​CTC​AG
Human *Cxcl9*	GTG​AGA​AAG​GGT​CGC​TGT​TC	GCT​GAC​CTG​TTT​CTC​CCA​CT
Human *Il1-ra*	AAC​ATC​ACT​GAC​CTG​AGC​GA	GCA​TAT​TGG​TGA​GGC​TGA​CG
Human *Il-1β*	GGA​GAA​TGA​CCT​GAG​CAC​CT	GGA​GGT​GGA​GAG​CTT​TCA​GT
Human *Il-10*	CAT​CAA​GGC​GCA​TGT​GAA​CT	CCA​CGG​CCT​TGC​TCT​TGT​TT
Human *Cox2*	TAC​CCT​CCT​CAA​GTC​CCT​GA	ACT​GCT​CAT​CAC​CCC​ATT​CA
Human *Gapdh*	AGG​TCG​GAG​TCA​ACG​GAT​TT	ATC​GCC​CCA​CTT​GAT​TTT​GG

### Western blot

The tissues were lysed using lysis buffer (Beyotime, P0013, China) to extract proteins. The protein samples were separated by SDS-PAGE and then transferred onto polyvinylidene difluoride (PVDF) membranes (GE, 10600023) via electroblotting. The proteins were probed with antibodies against SDH, COX-2, HIF-α, GAPDH (Yeasen, 30201ES20, China), NLRP3, ASC, AIM2, MLKL (Merck, MABC604, United States). Reactivity was determined using HRP-conjugated secondary antibodies (Yeasen, China). The proteins were visualized by super ECL detection reagent (Yeasen, 36208ES60, China).

### Sample preparation for proteome

For each treatment group, BMDMs were collected and washed in PBS and subjected to global protein extraction using 8M Urea (PH 8.0) containing protease inhibitor (phenylmethanesulfonyl fluoride, PMSF), followed by 3 min of sonication (3s on, 3s off, amplitude 25%). Then the protein concentration was obtained through Bradford quantification assay and 100 μg protein was digested overnight following filter-acid sample preparation (FASP) method with 3.5ug trypsin in 50 mM ammonium acid carbonate (PH 8.0) overnight at 37°C. Finally, the purified peptides were acquired after extraction with 50% acetonitrile (ACN) and 0.1% formic acid (FA) following desalination in two layers of Empore 3M C18 disk with 2 mg packing (3 μm, 150 Å, Agela) in a pipet tip and dried in a vacuum concentrator (Thermo Scientific).

### Liquid chromatography tandem mass spectrometry (LC-MS/MS) analysis of peptide mixture

Proteome analysis was processed on a nanoElute-HPLC System (Bruker Daltonics) coupled with a hybrid trapped ion mobility spectrometry quadrupole times-of-flight mass spectrometer (TIMS-TOF Pro Bruker Daltonics, Billerica, MA) via a Captive Spray nano-electrospray ion source. Peptide mixture was re-dissolved in solution A (0.1% FA) and loaded onto the analytical column (75 μm i.d. × 25 cm) and separated with a 60 min gradient (2–22% solvent B (ACN with 0.1% formic acid) for 45 min, 22–37% B for 5 min, 37–80% B for 5 min, and then 80% B for 5 min) at a flow rate of 600 nL/min. The MS analysis was performed by scanning 100–1700 m/z in positive electrospray mode. The accumulation and ramp time were set as 100 ms each. Survey full-scan MS spectra (m/z 100–1,700) were obtained. The ion mobility was scanned from 0.7 to 1.3 Vs/cm^2^. The overall acquisition cycle of 1.16s comprised one full TIMSMS scan and 10 parallel accumulation-serial frag-mentation (PASEF) MS/MS scans. During PASEF MSMS scanning, the collision energy was ramped linearly as a function of the mobility from 59 eV at 1/K0 = 1.6 Vs/cm^2^ to 20 eV at 1/K0 = 0.6 Vs/cm^2^.

### Proteome identification and quantification with maxquant-based database searching

MS raw files were searched against the mouse Refseq protein database (version 20171126) in the National Center for Biotechnology Information (NCBI) using Maxquant (Version 1.5.3.30). Trypsin was selected as the proteolytic enzyme, and two missed cleavages sites were allowed. The mass tolerance was 20 ppm for precursor and 0.5 Da for production. The oxidation of Methionine and N-acetylation were set as the variable modifications. The false discovery rates of the peptide-spectrum matches (PSMs) and proteins were set to less than 1%. Proteins with at least 1 unique peptide were selected for further analysis. For the proteome quantification, the intensity-based absolute quantification (iBAQ) value was extracted from MaxQuant results and subjected to FOT calculation. FOT was defined as protein’s iBAQ divided by the total iBAQ of all identified proteins in one experiment. Finally, FOT was multiplied by 10^6^ for easy presentation. All data generated in this study, including the raw files and quantitative data matrix of proteomes, have been deposited to iproX (https://111.198.139.98/page/home.html) with accession number IPX0009840000.

### Function enrichment analysis

Gene function enrichment analysis was performed with the R Bioconductor package “clusterProfiler” (R package v3.14.3) based on Gene Ontology (GO) database or Kyoto Encyclopedia of Genes and Genomes (KEGG) database. Enrichment scores were determined using Fisher’s exact test.

### Statistical analyses

Data are presented as mean ± SEM. Two and more independent experiments were performed for each statistic. Statistical analysis was performed by Student’s *t* test or ANOVA.

## Data Availability

The datasets presented in this study can be found in online repositories. The names of the repository/repositories and accession number(s) can be found in the article/Supplementary Material. The Project ID is IPX0009840000. The ProteomeXchange ID is PXD056595.

## Supplementary material

SUPPLEMENTARY FIGURE S1 Construction of Mlkl knockout mice **(A)** Schematic diagram of Mlkl knockout. **(B)** Genotype confirmation of WT and Mlkl knockout mice.
