# Supplementary figures and images for: The critical role of MLKL in hemorrhagic stroke and the therapeutic potential of its associated protein network
Source: Front Cell Dev Biol. 2025 Jan 20;12:1509877. doi: 10.3389/fcell.2024.1509877 (PMC11788306; doi:10.3389/fcell.2024.1509877)

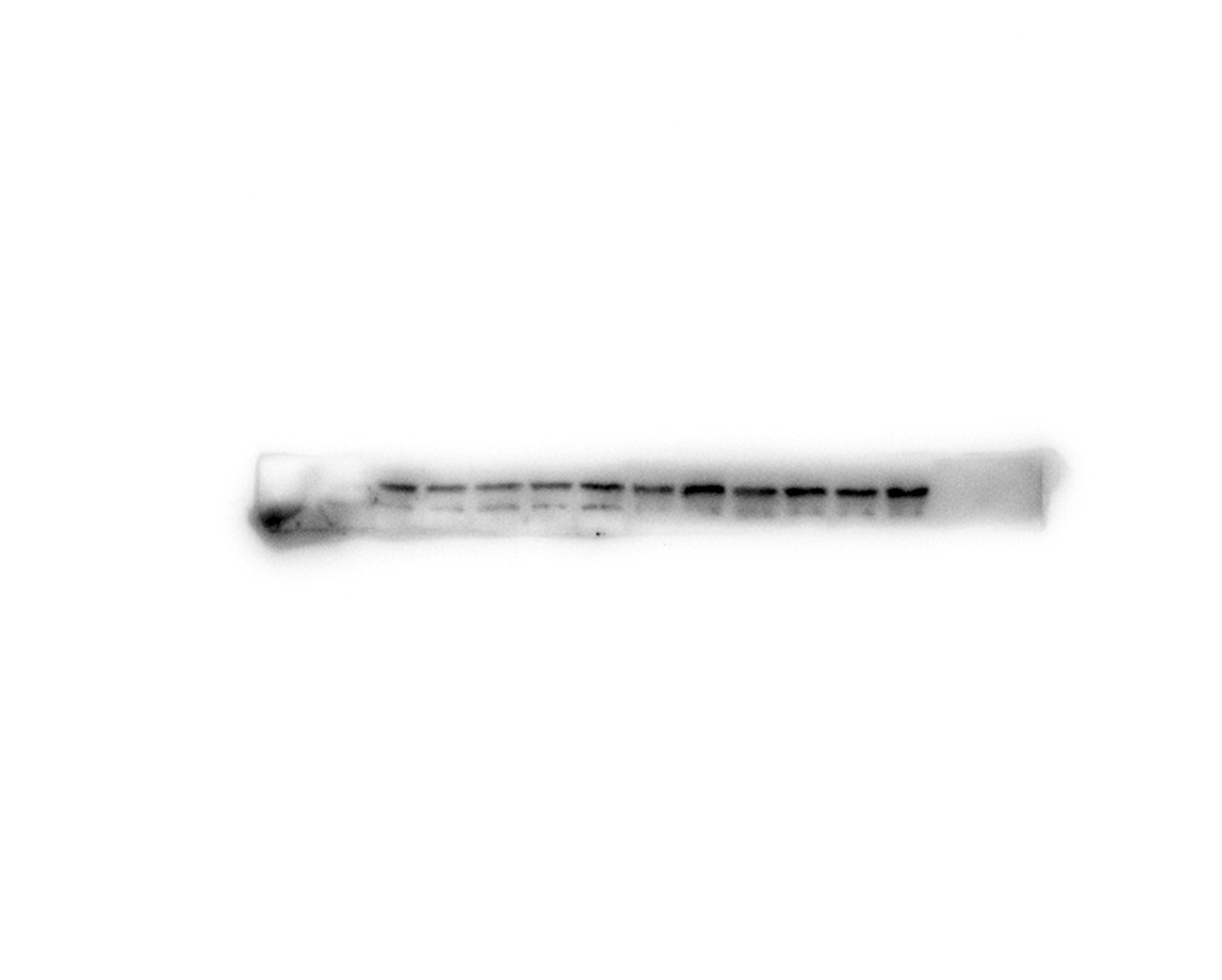

Supplement: Supplementary file 1 [file DataSheet1.zip › AIM2-F.tif]

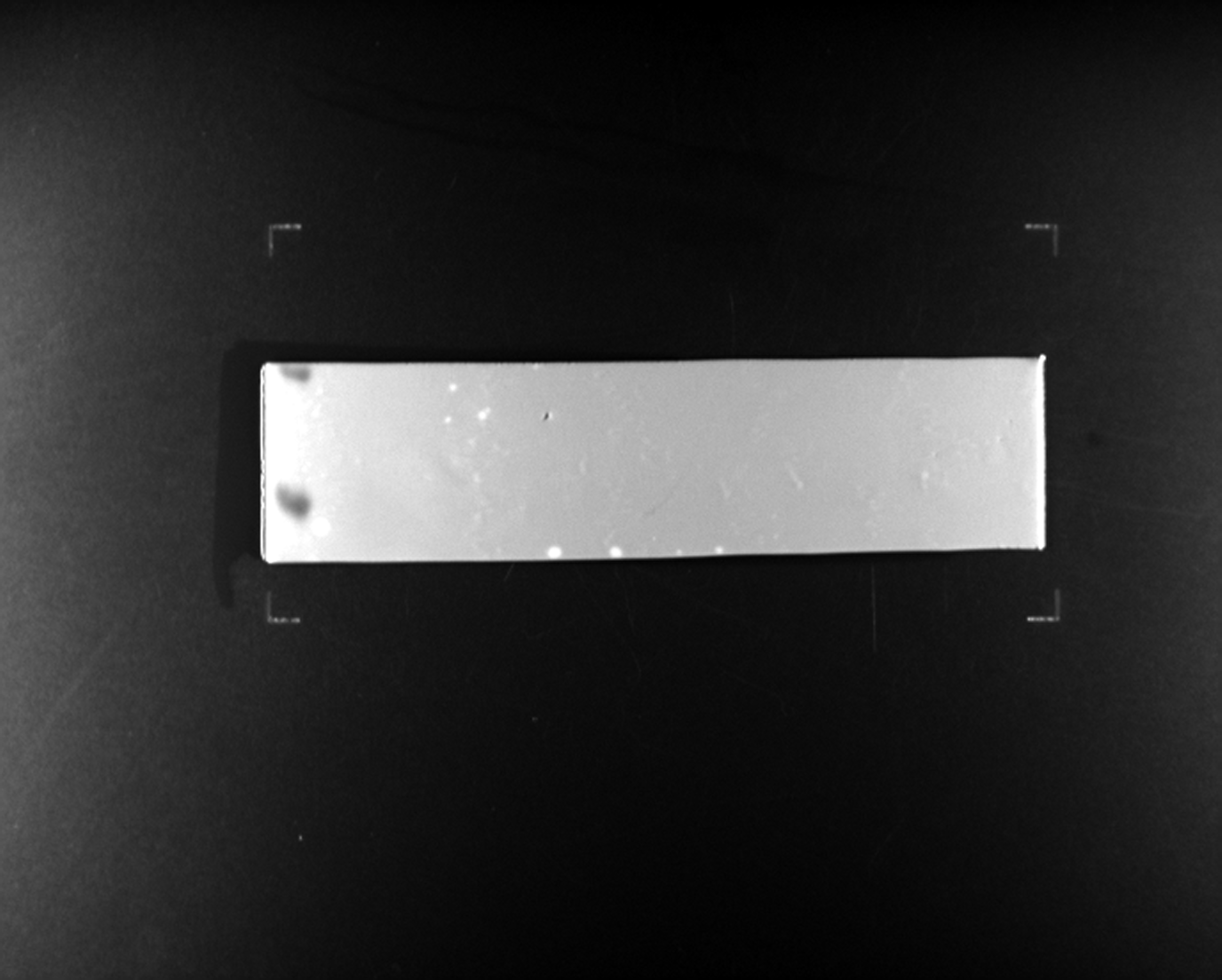

Supplement: Supplementary file 1 [file DataSheet1.zip › ASC-B.tif]

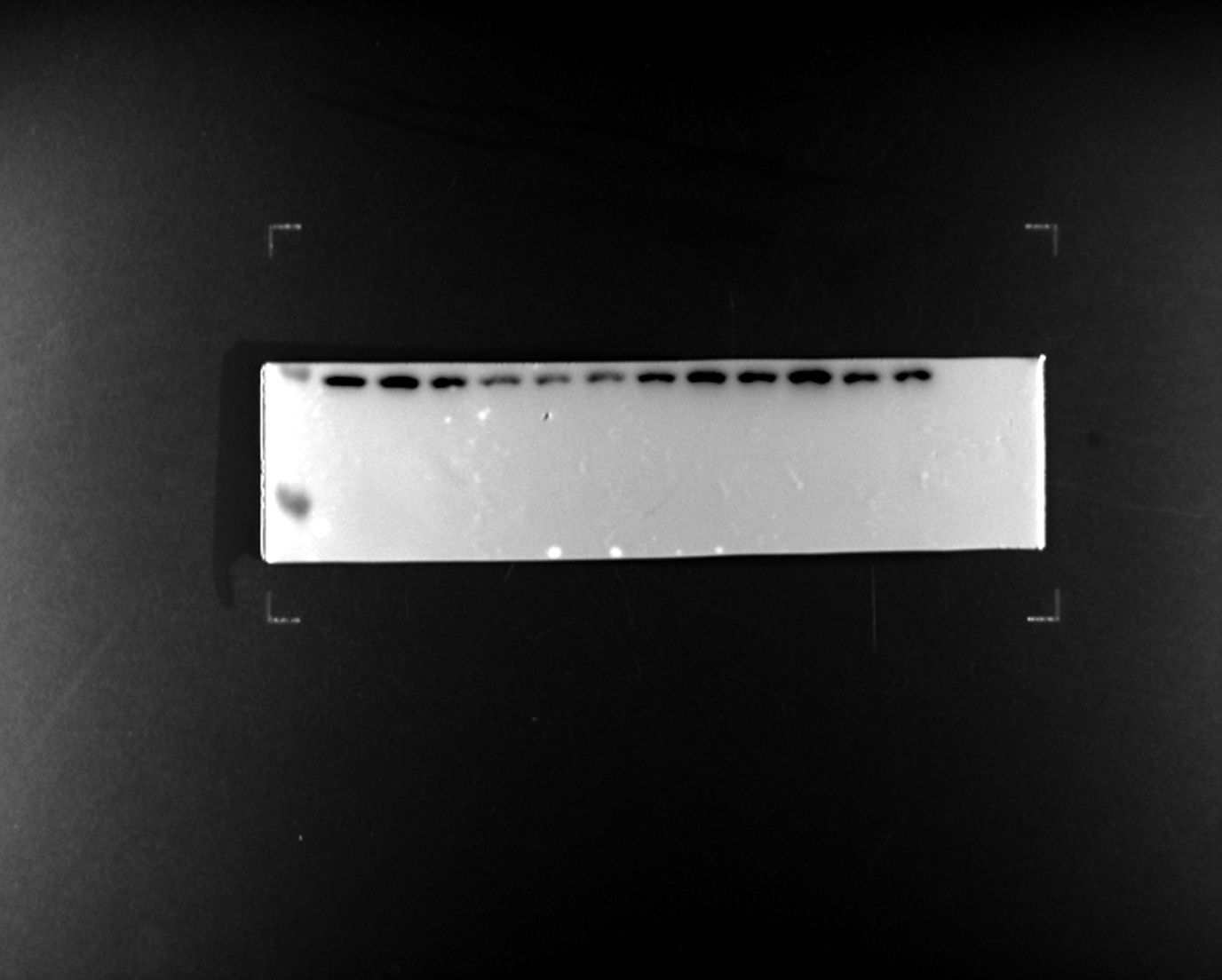

Supplement: Supplementary file 1 [file DataSheet1.zip › ASC-B+F.tif]

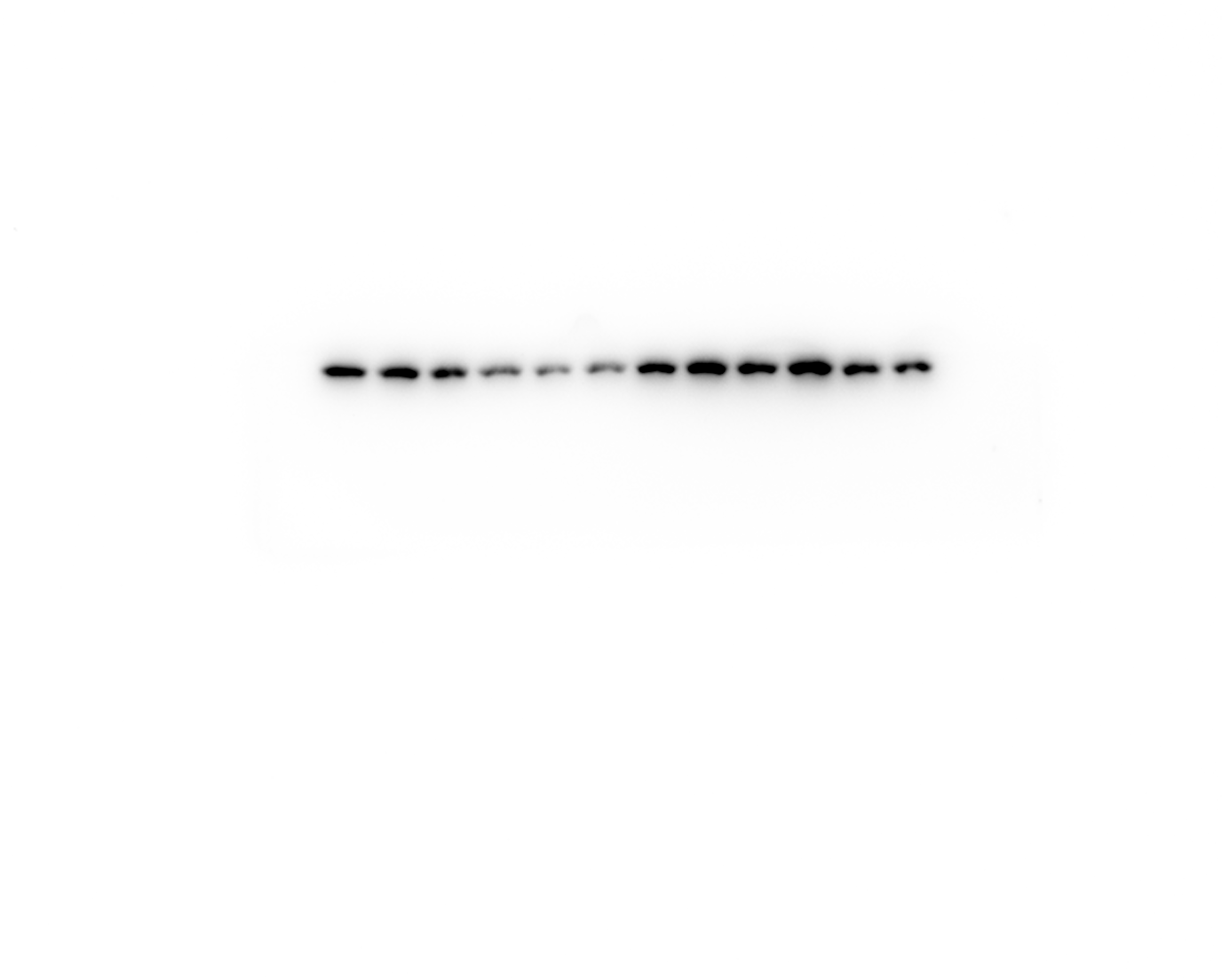

Supplement: Supplementary file 1 [file DataSheet1.zip › ASC-F.tif]

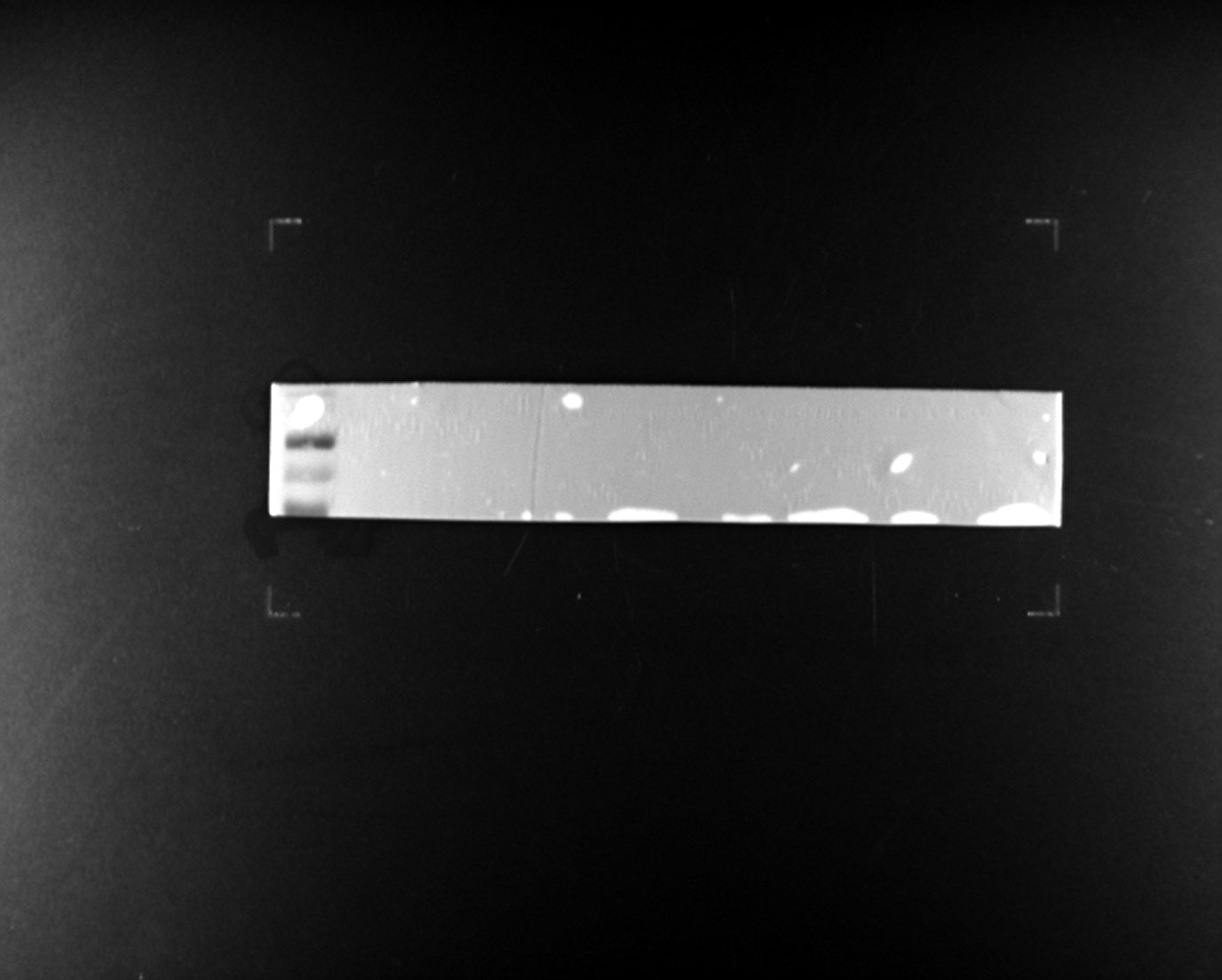

Supplement: Supplementary file 1 [file DataSheet1.zip › COX2-B.tif]

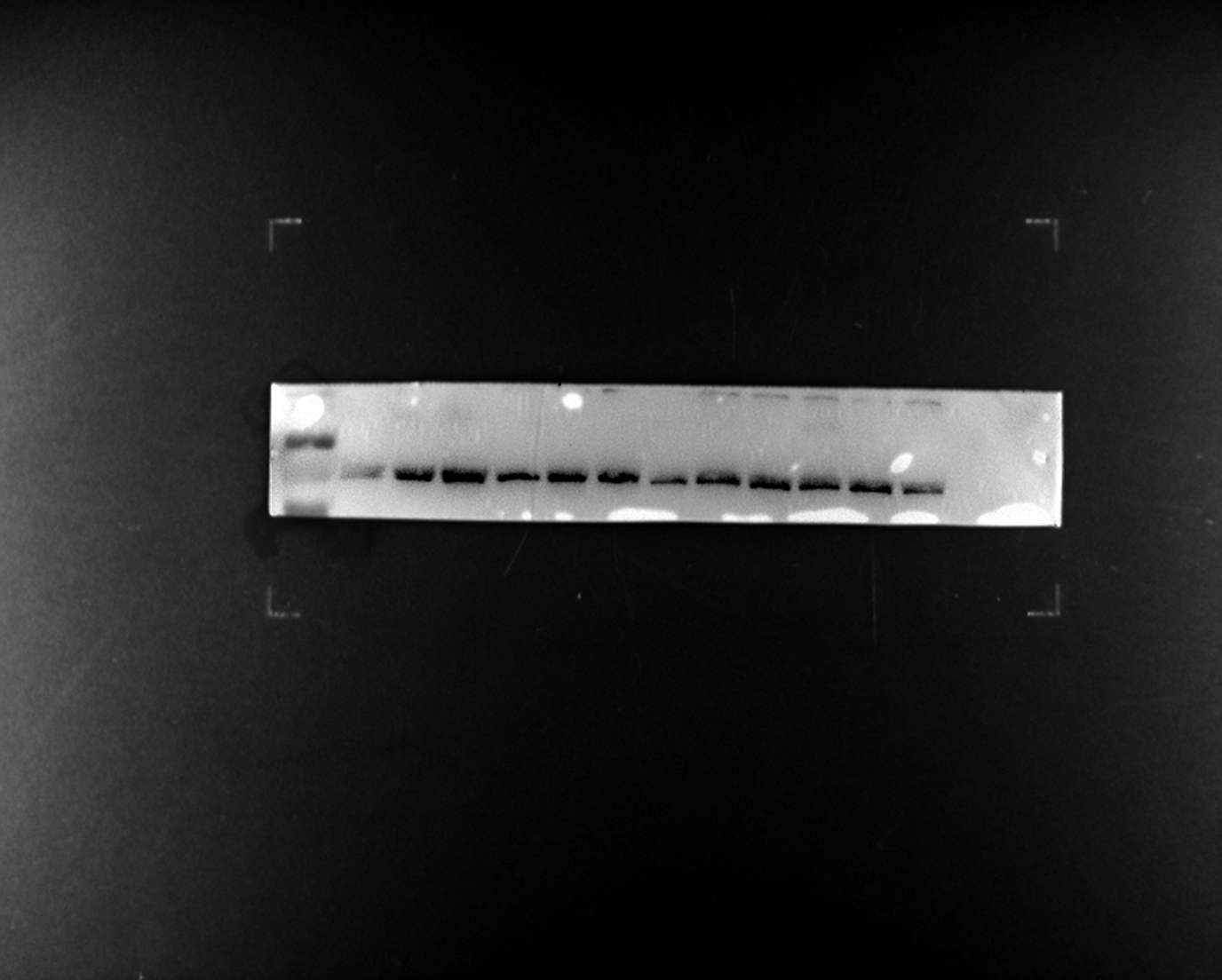

Supplement: Supplementary file 1 [file DataSheet1.zip › COX2-B+F.tif]

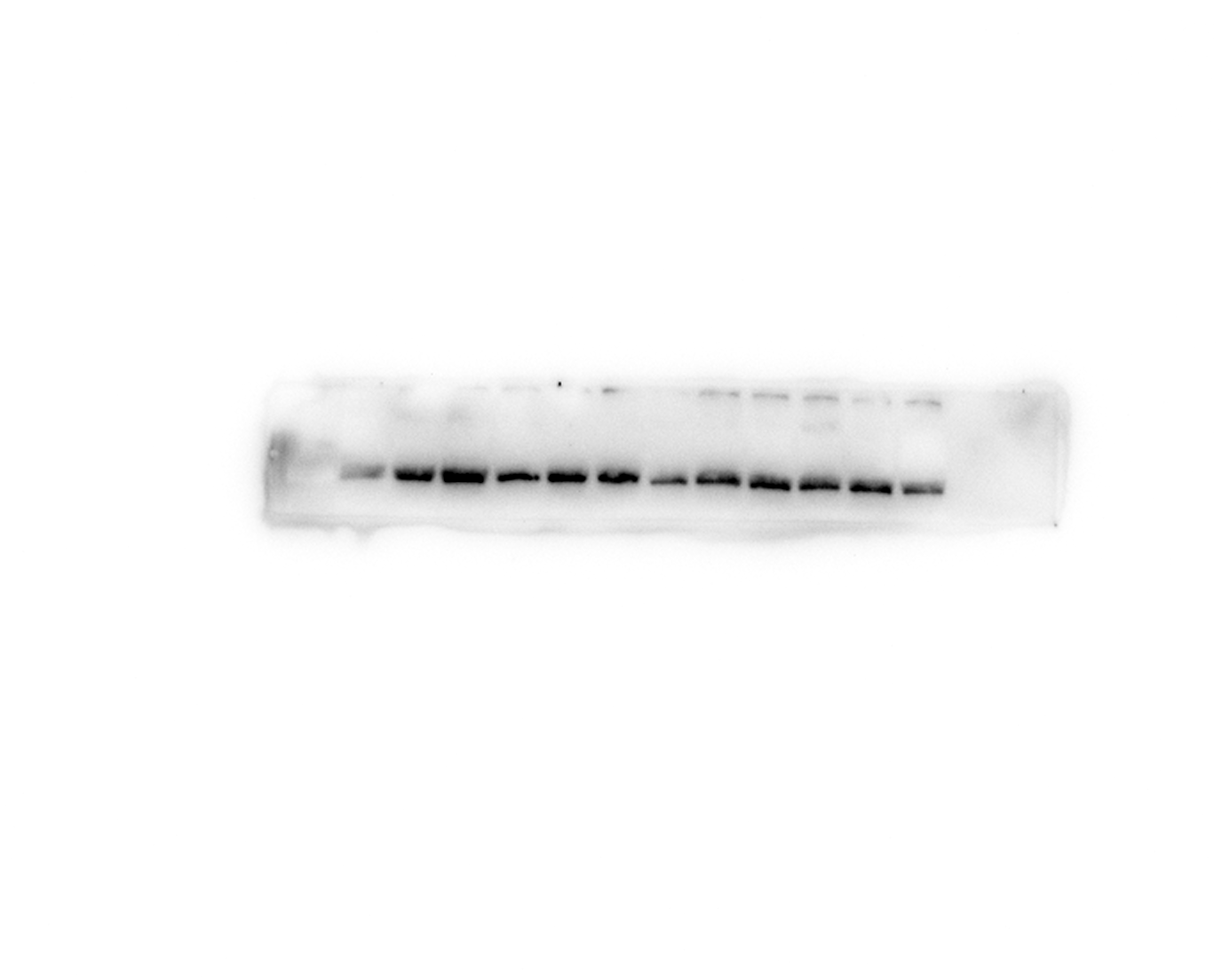

Supplement: Supplementary file 1 [file DataSheet1.zip › COX2-F.tif]

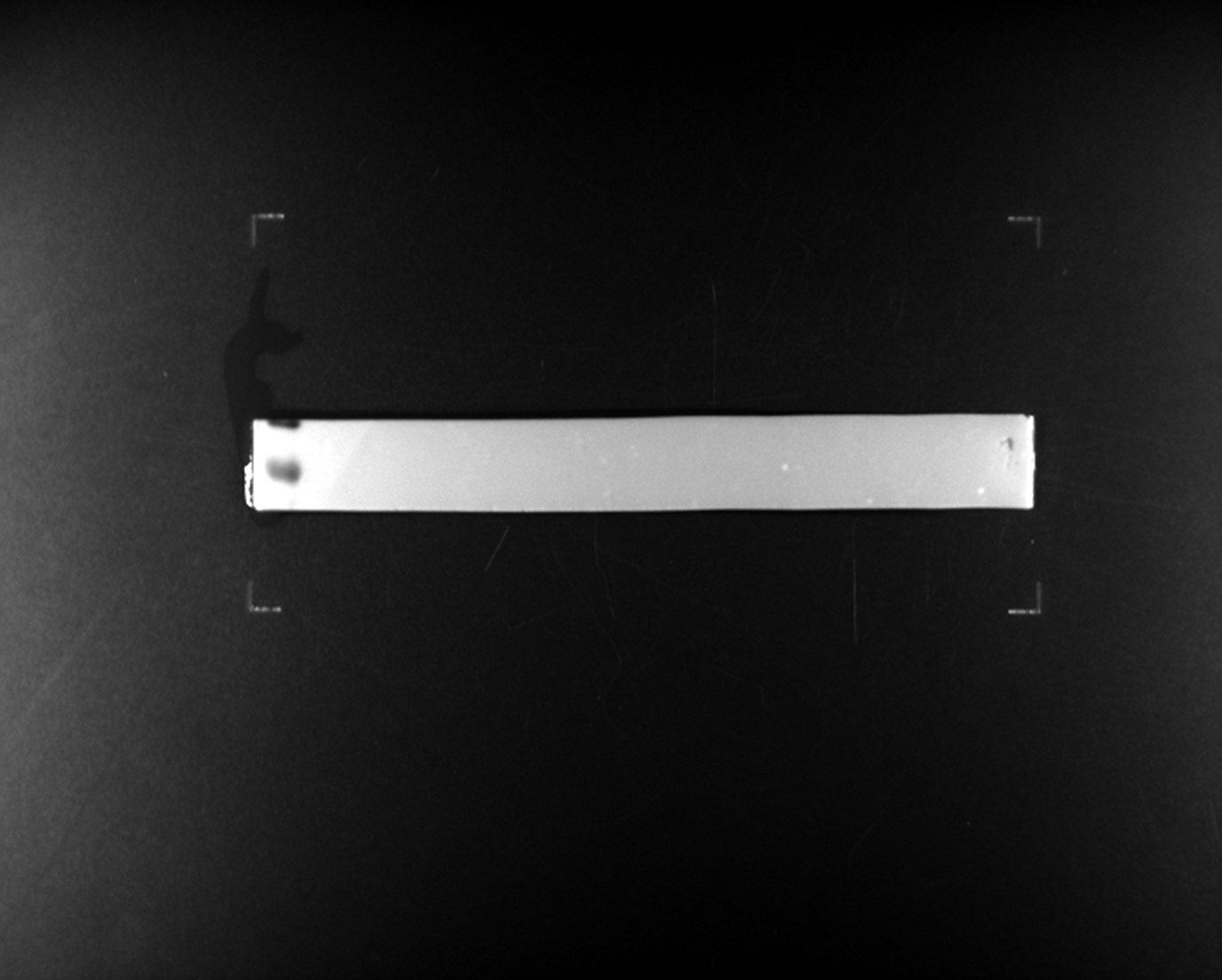

Supplement: Supplementary file 1 [file DataSheet1.zip › GAPDH-B.tif]

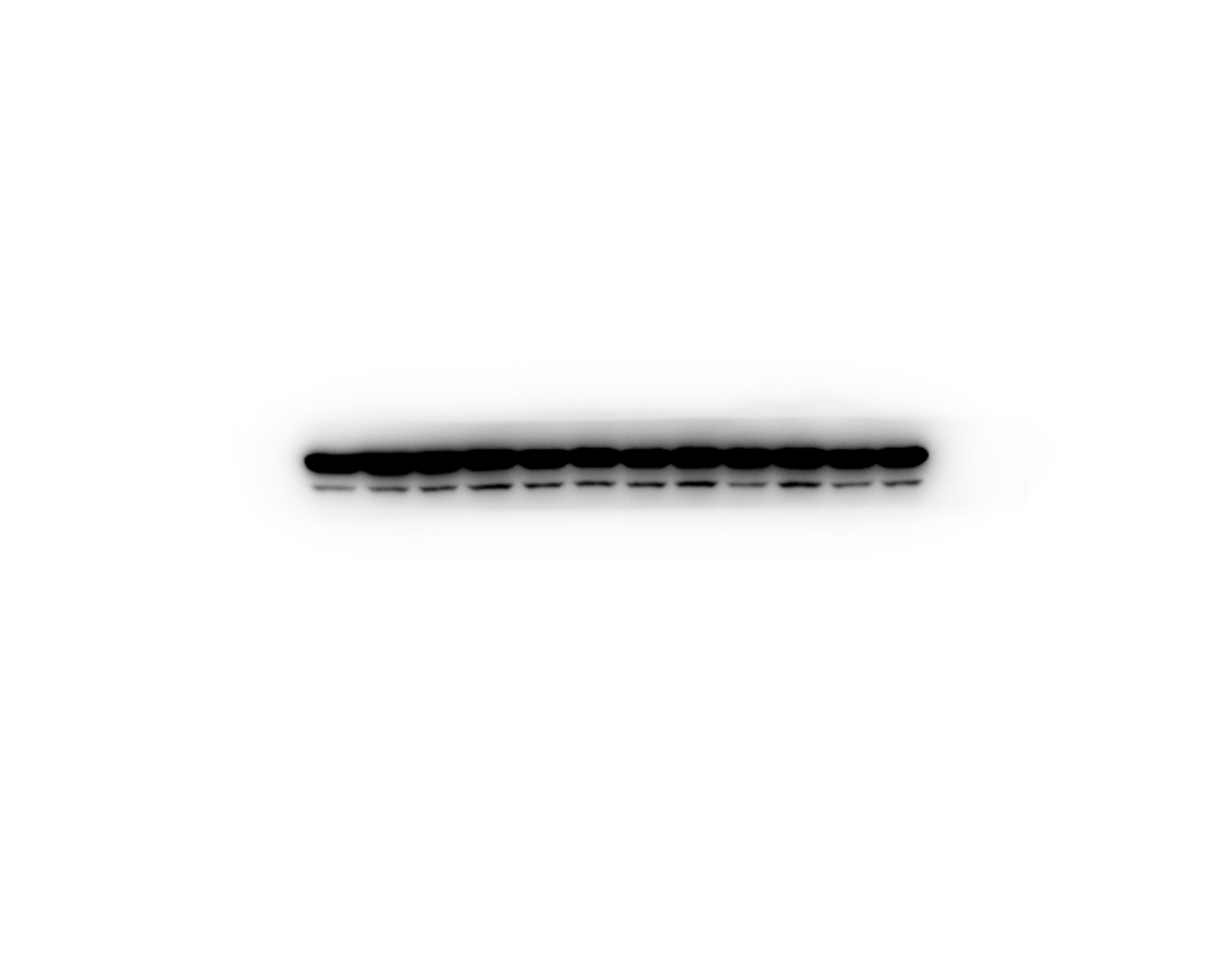

Supplement: Supplementary file 1 [file DataSheet1.zip › GAPDH-F.tif]

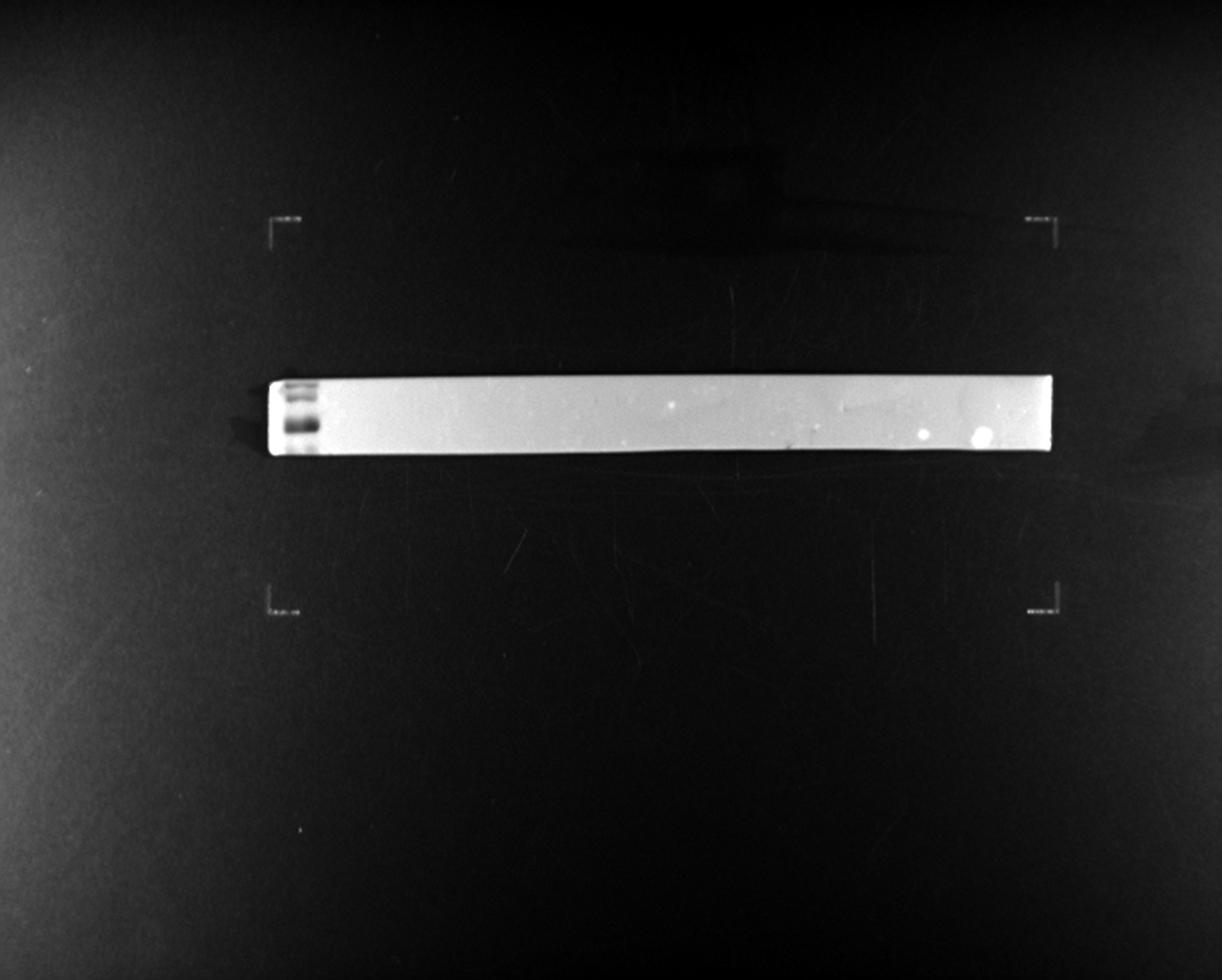

Supplement: Supplementary file 1 [file DataSheet1.zip › HIF1a-B.tif]

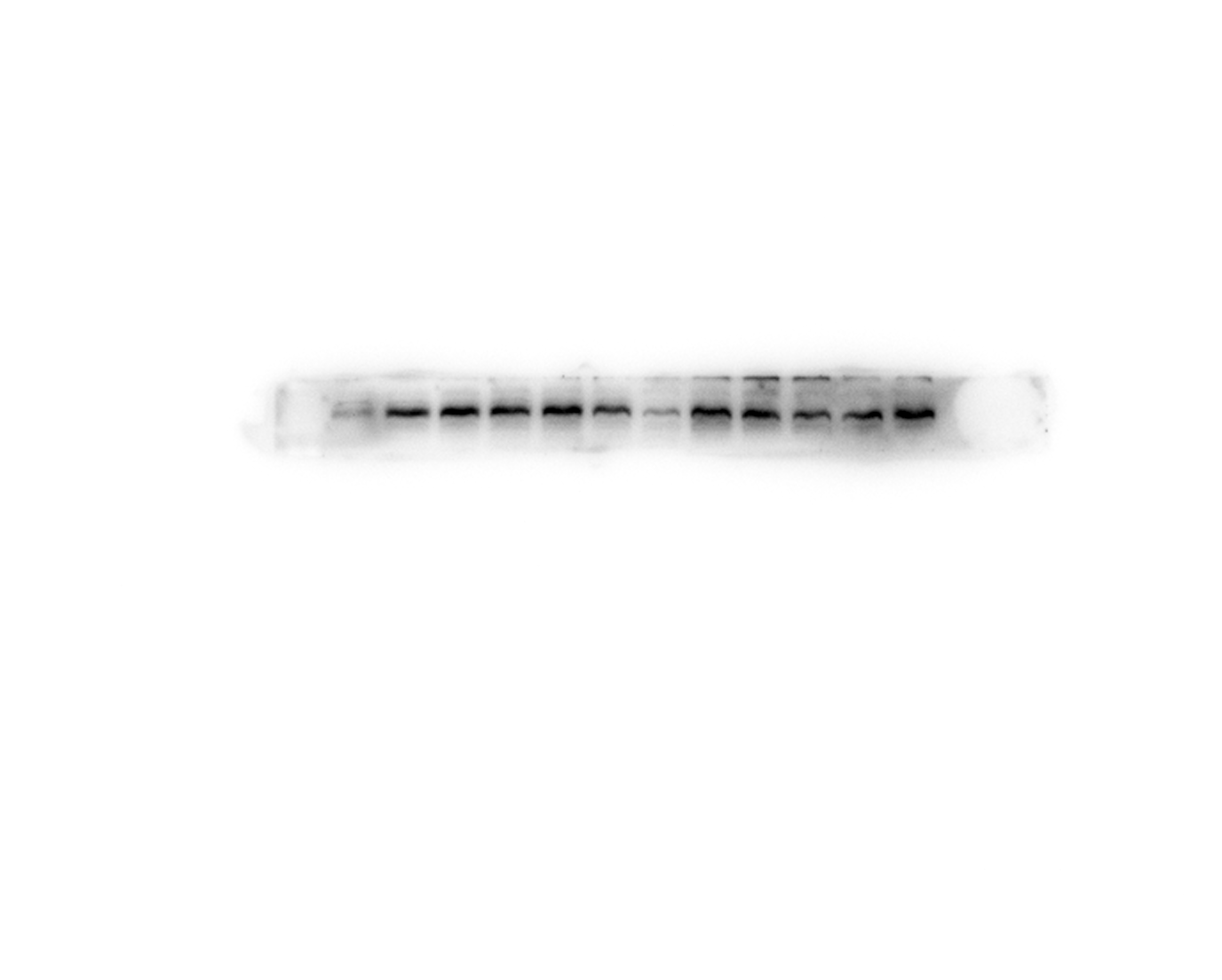

Supplement: Supplementary file 1 [file DataSheet1.zip › HIF1a-F.tif]

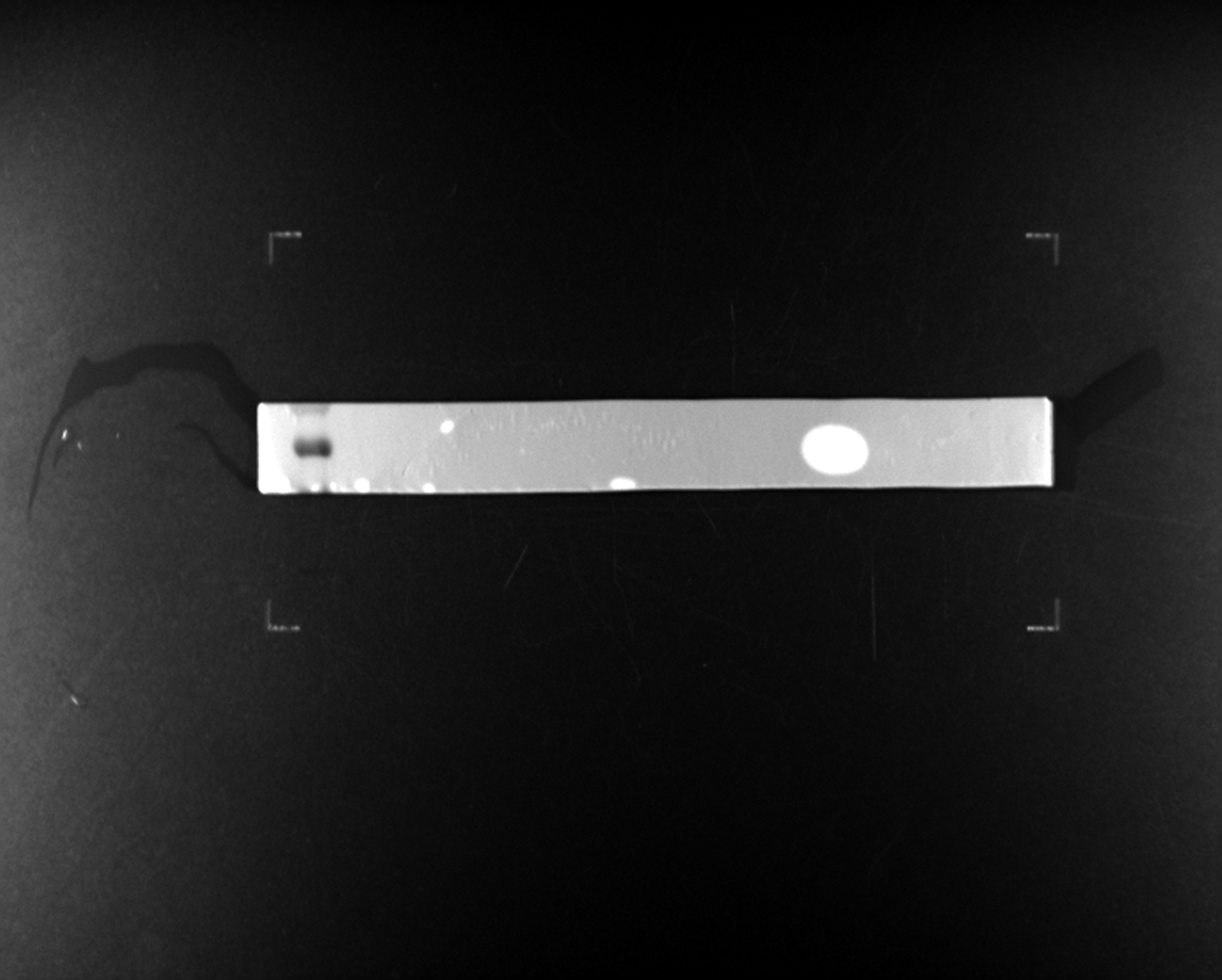

Supplement: Supplementary file 1 [file DataSheet1.zip › MLKL-B.tif]

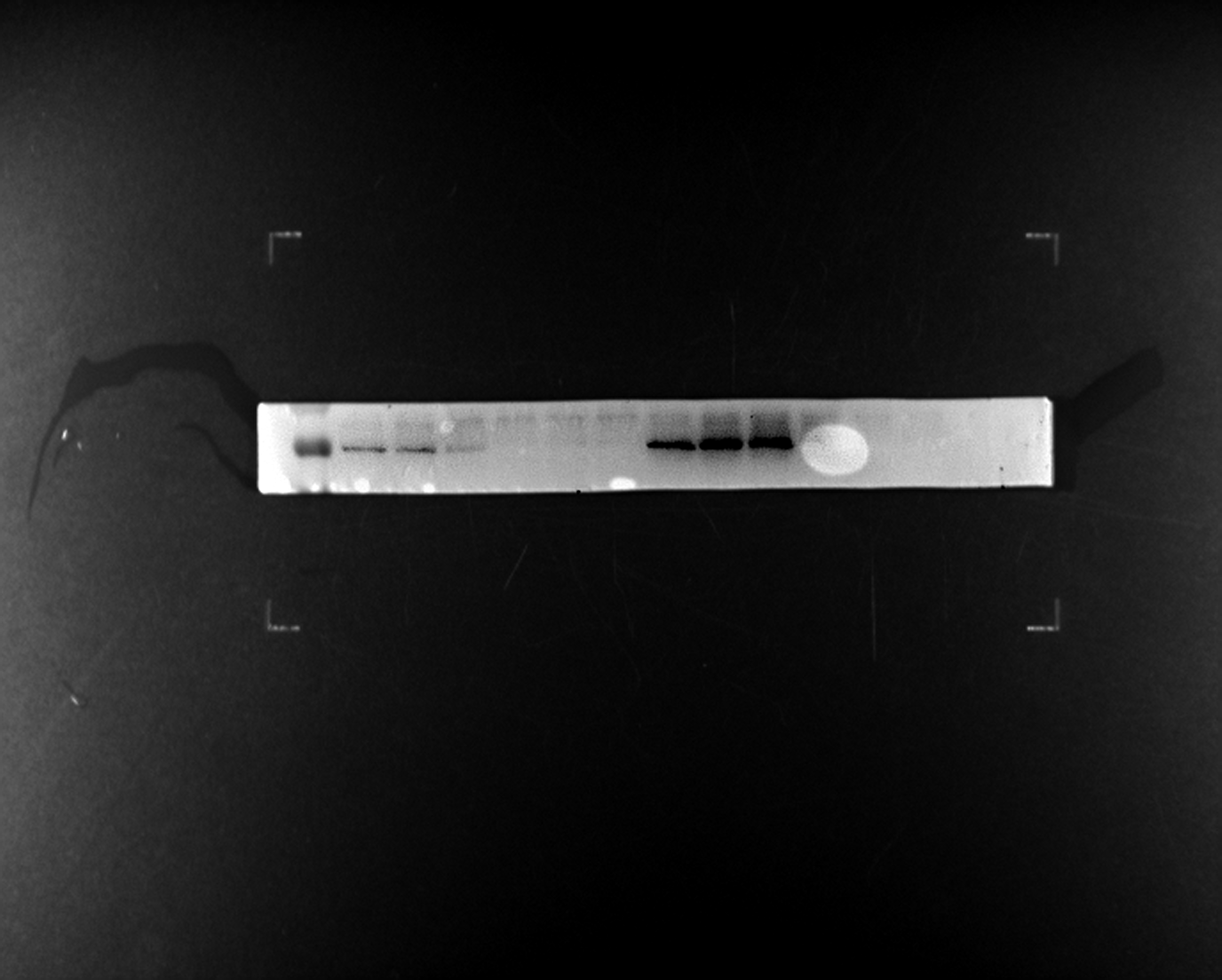

Supplement: Supplementary file 1 [file DataSheet1.zip › MLKL-B+F.tif]

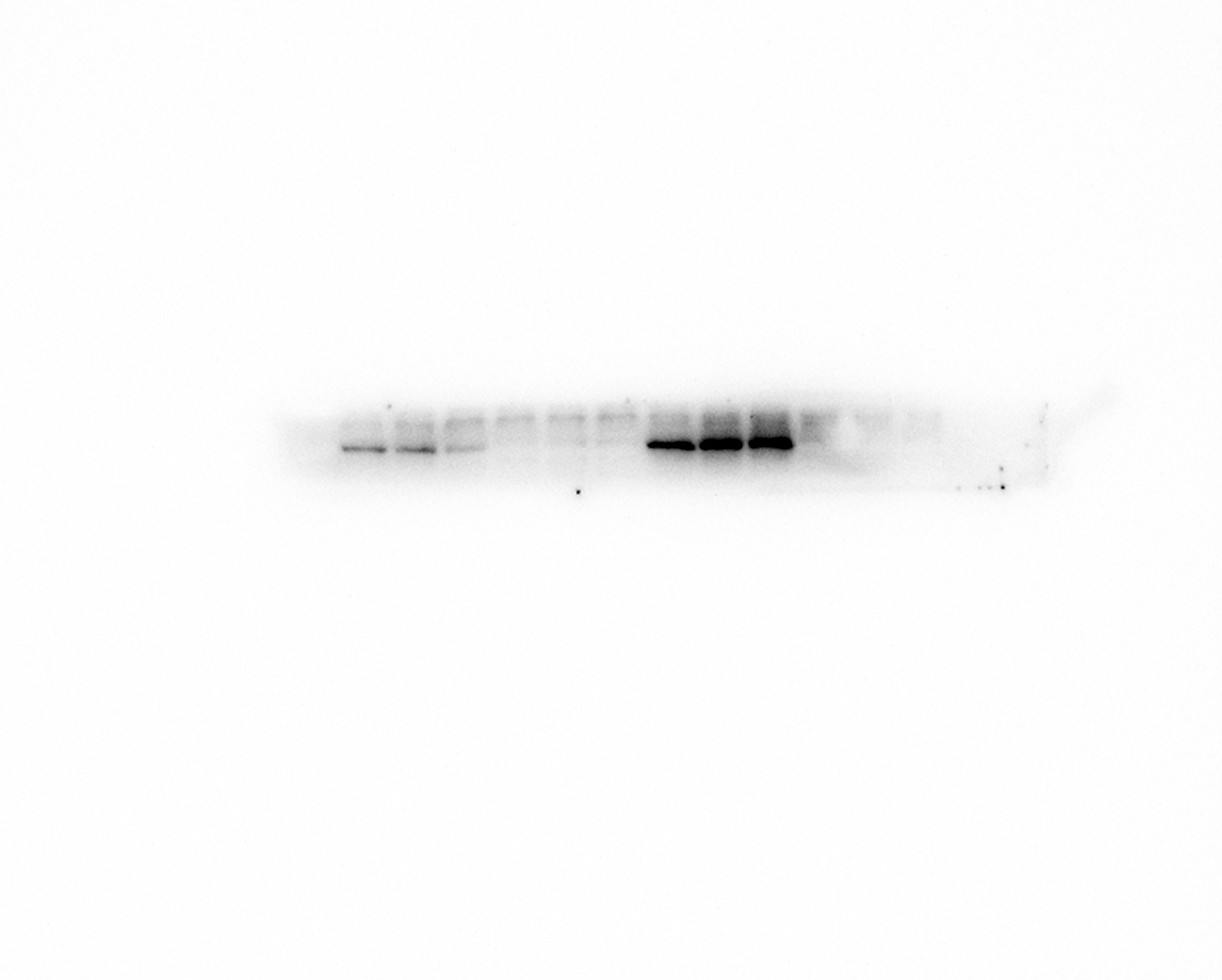

Supplement: Supplementary file 1 [file DataSheet1.zip › MLKL-F.tif]

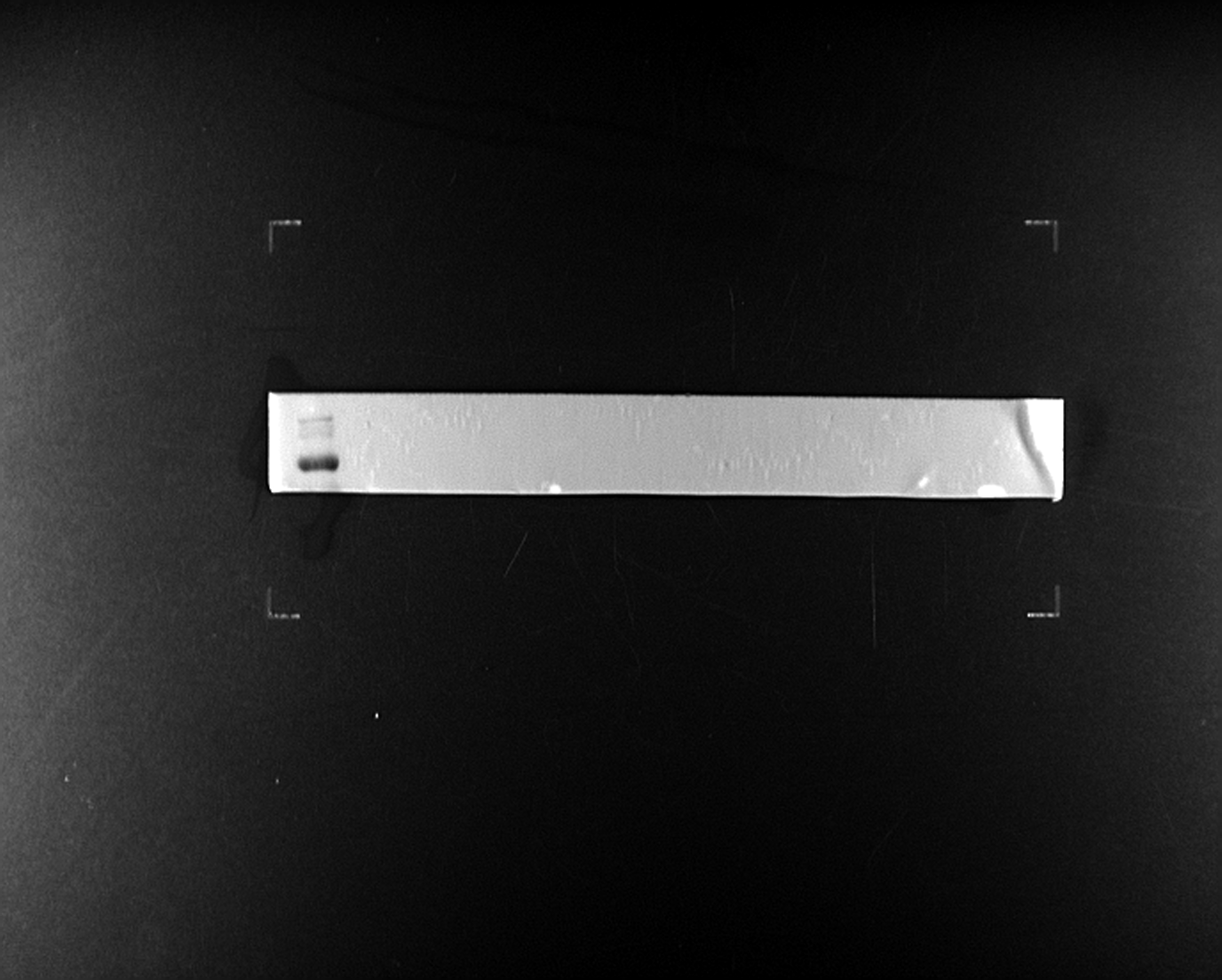

Supplement: Supplementary file 1 [file DataSheet1.zip › NLRP3-B.tif]

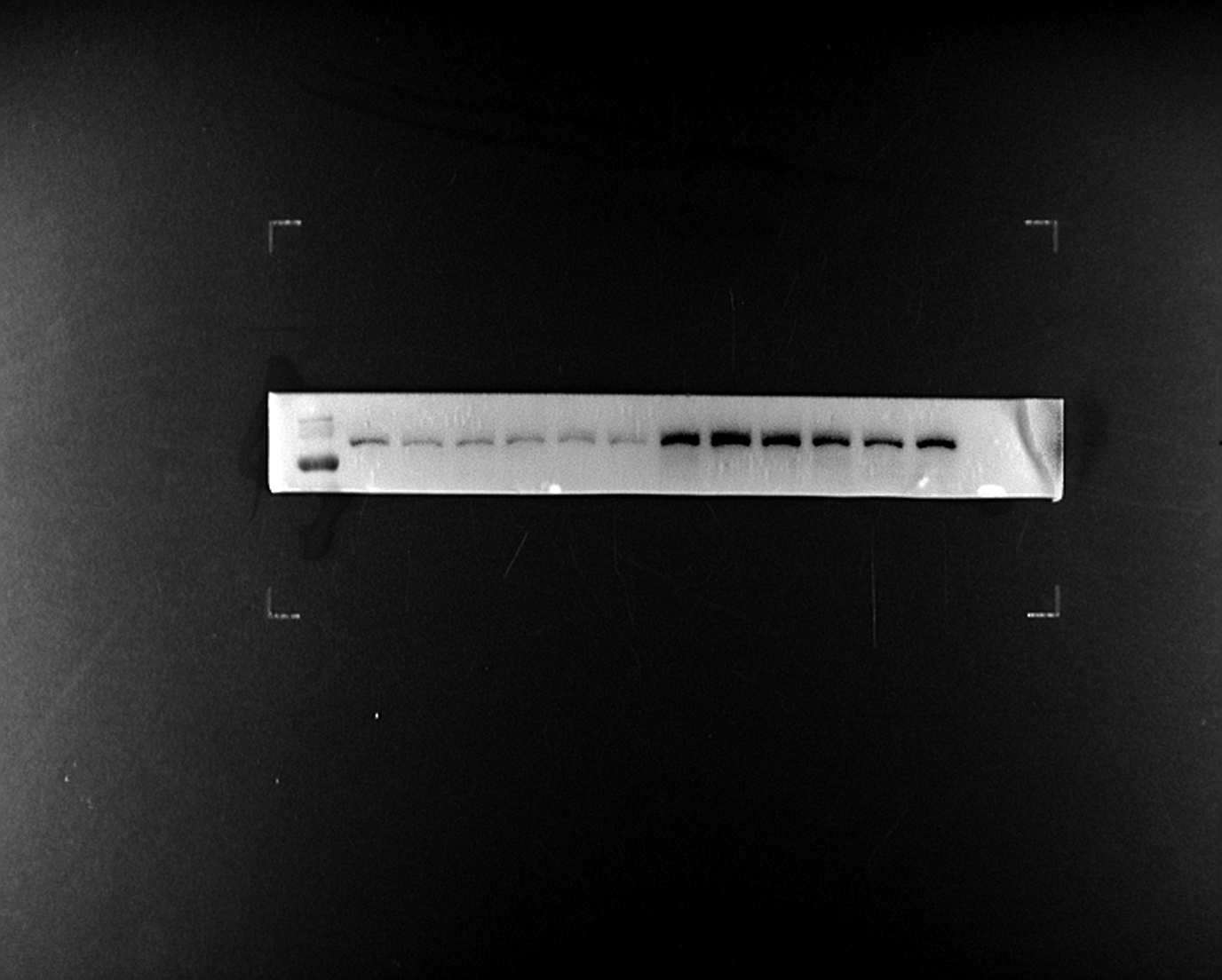

Supplement: Supplementary file 1 [file DataSheet1.zip › NLRP3-B+F.tif]

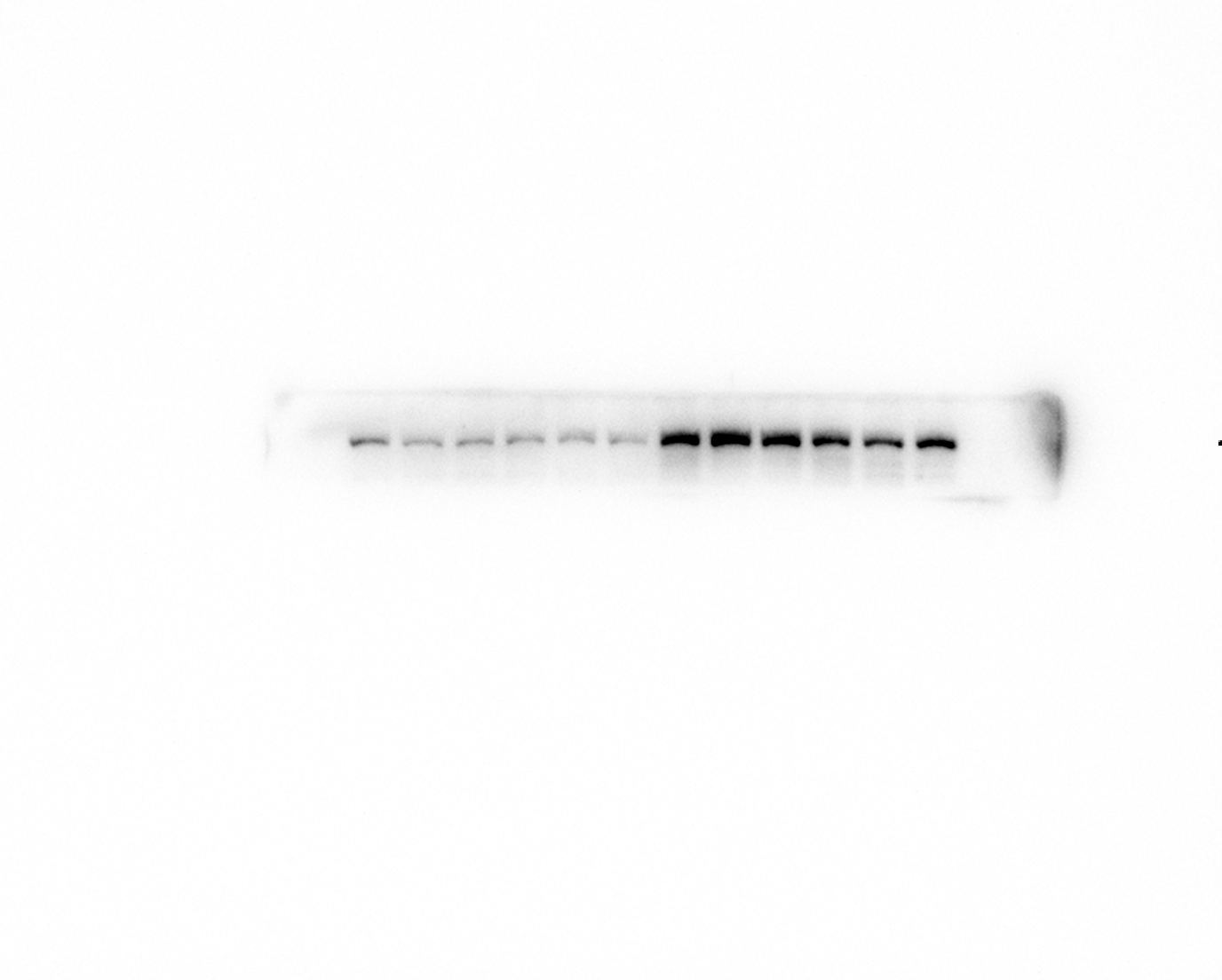

Supplement: Supplementary file 1 [file DataSheet1.zip › NLRP3-F.tif]

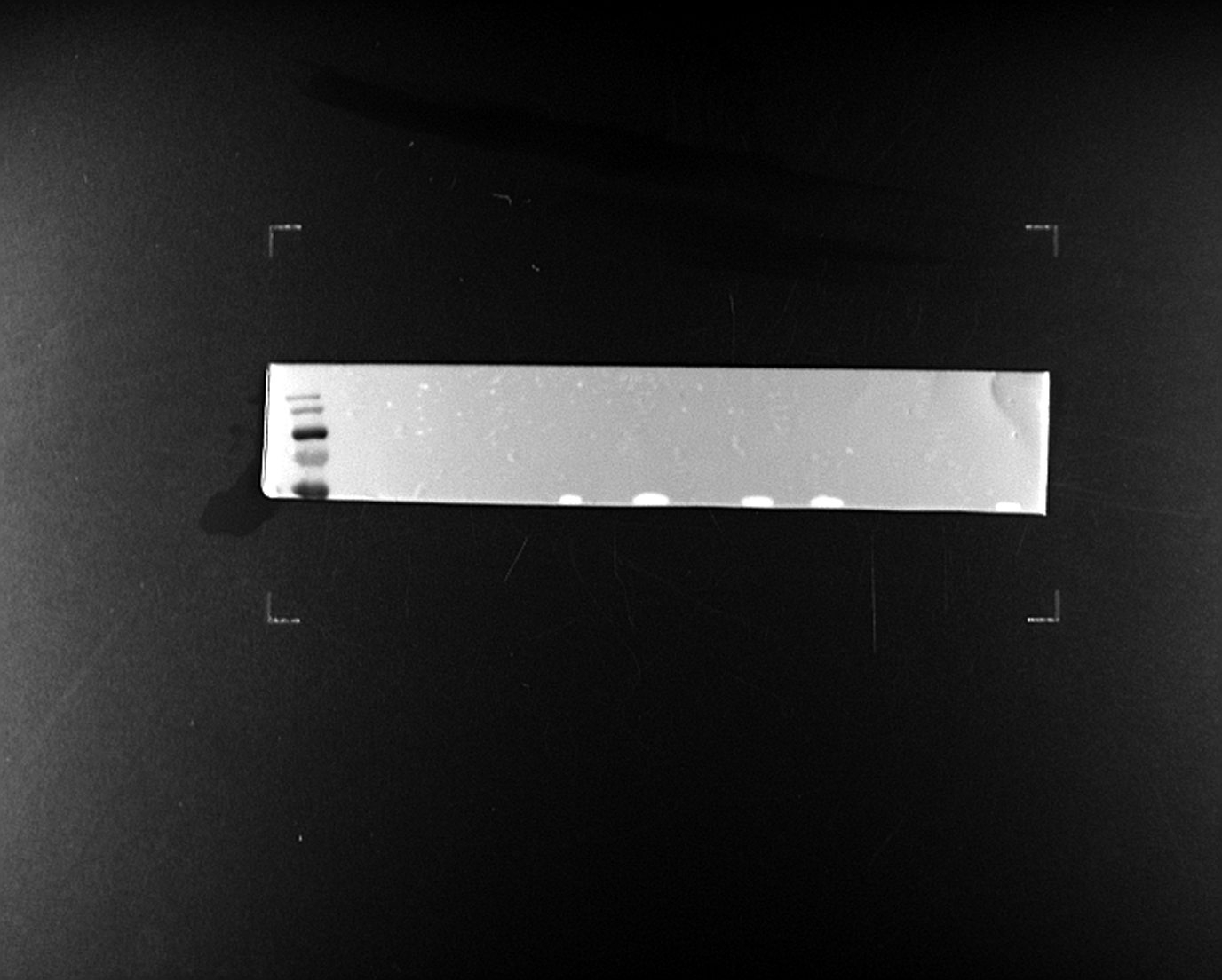

Supplement: Supplementary file 1 [file DataSheet1.zip › SDH-B.tif]

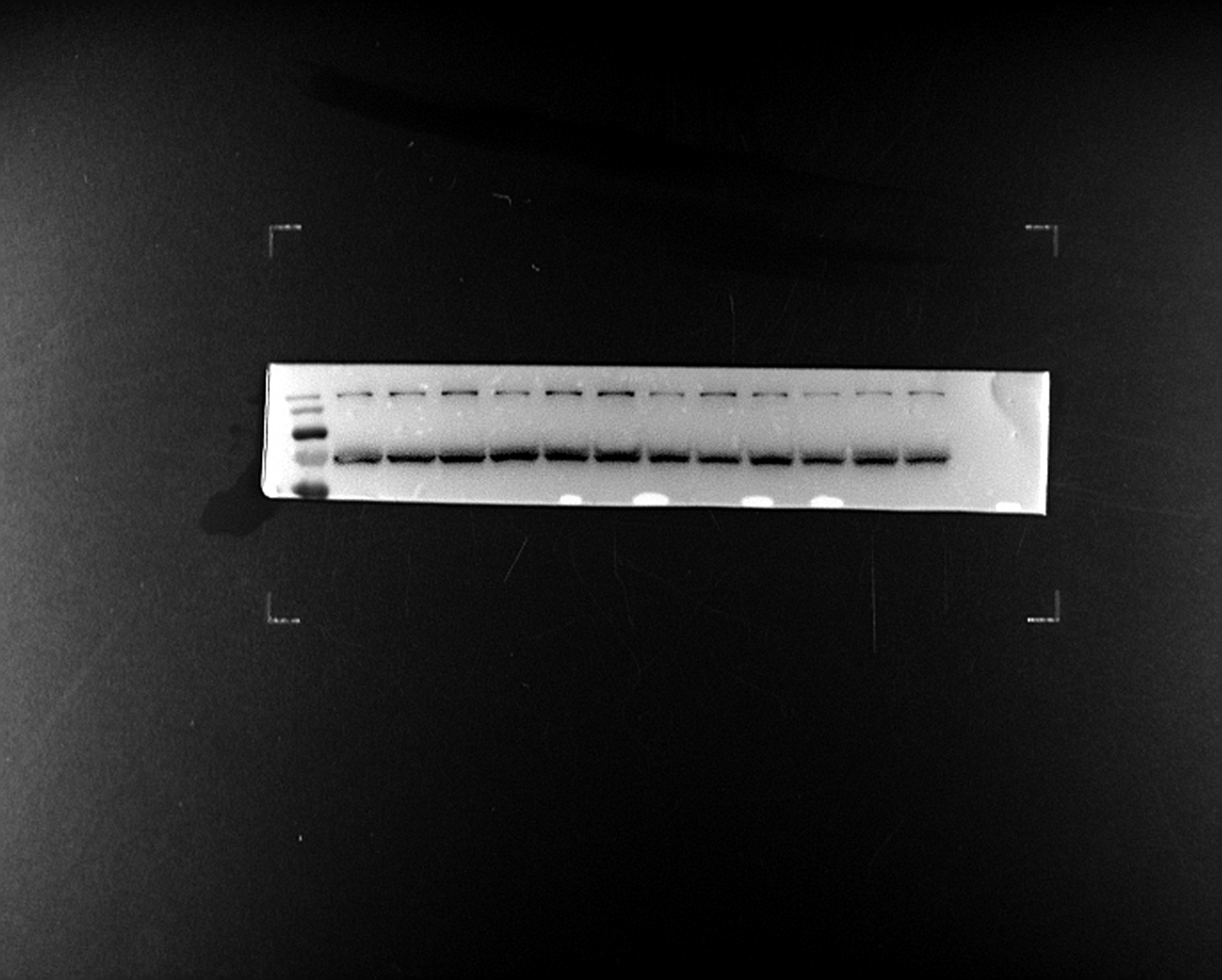

Supplement: Supplementary file 1 [file DataSheet1.zip › SDH-B+F.tif]

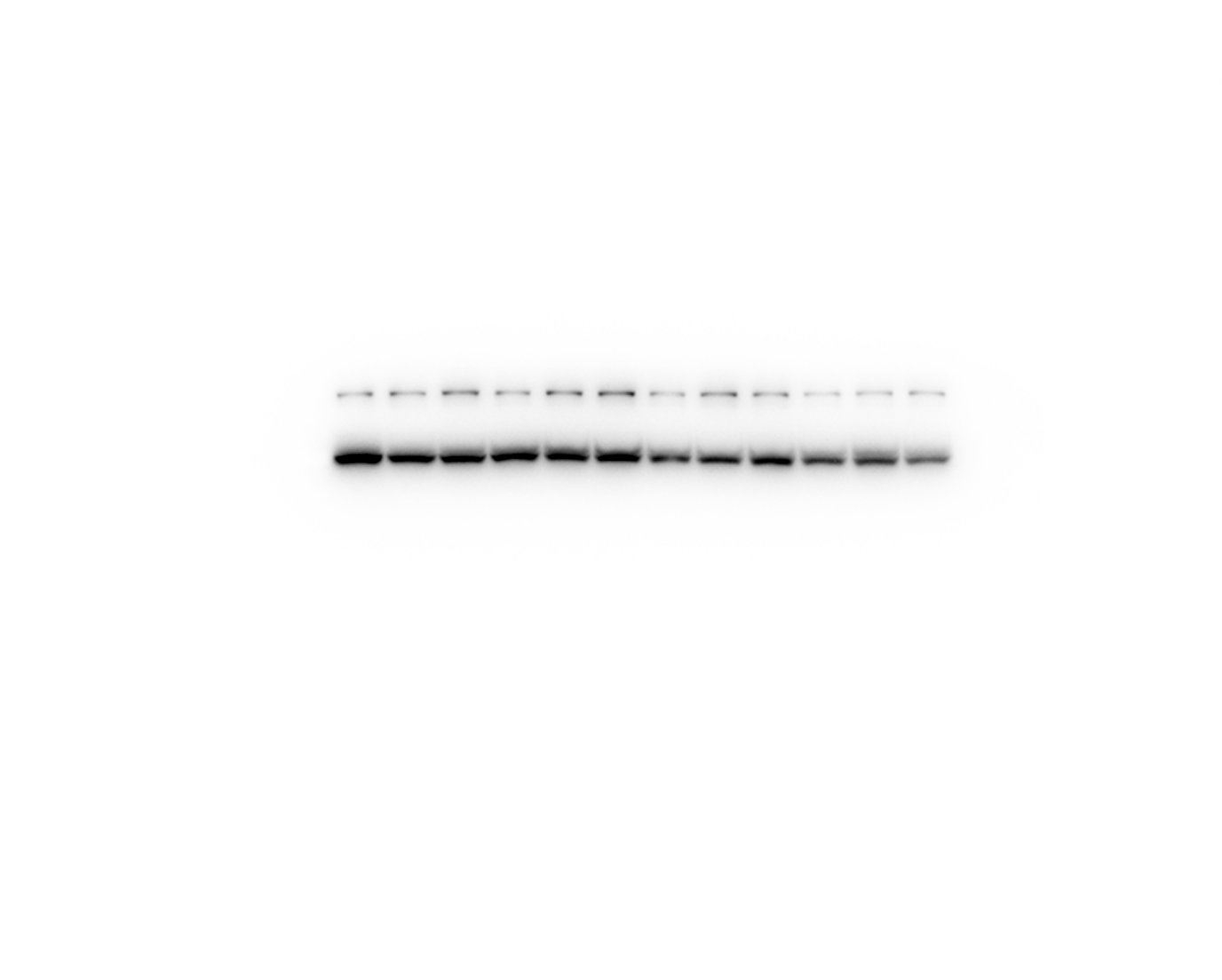

Supplement: Supplementary file 1 [file DataSheet1.zip › SDH-F.tif]

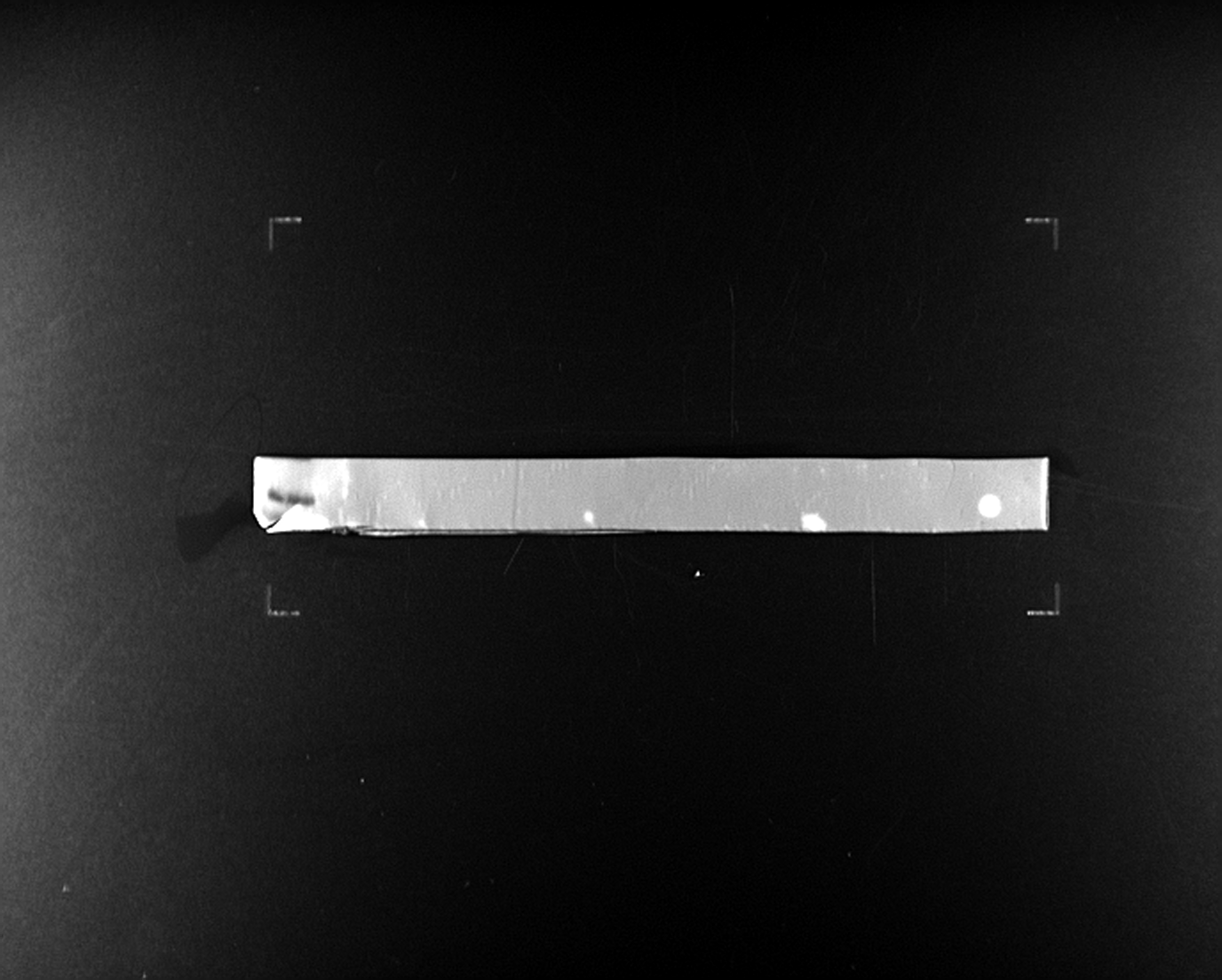

Supplement: Supplementary file 1 [file DataSheet1.zip › AIM2-B.tif]

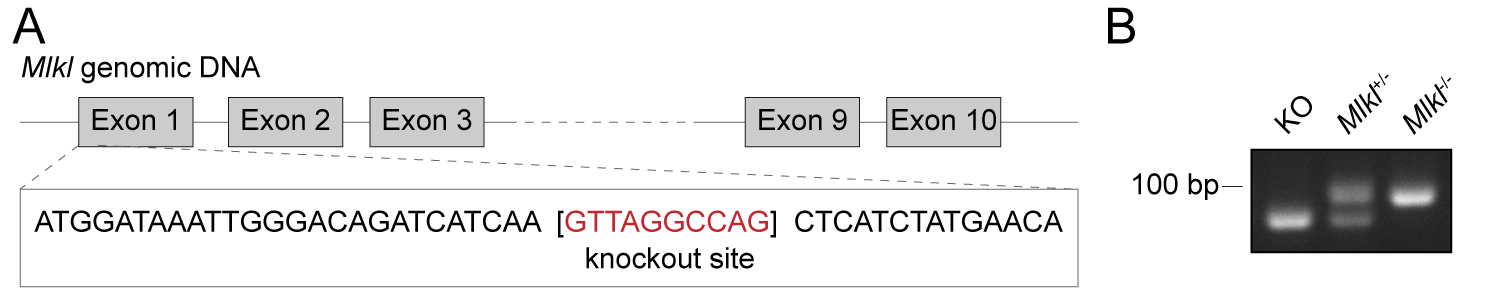

Supplement: Supplementary file 2 [file Image1.tif]
